# Non-invasive photobiomodulation treatment in an Alzheimer Disease-like transgenic rat model

**DOI:** 10.7150/thno.70756

**Published:** 2022-02-14

**Authors:** Luodan Yang, Chongyun Wu, Emily Parker, Yong Li, Yan Dong, Lorelei Tucker, Darrell W. Brann, Hung Wen Lin, Quanguang Zhang

**Affiliations:** 1 Department of Neurology, Louisiana State University Health Sciences Center, Shreveport, LA, 1501 Kings Highway, LA 71103 USA.; 2 Medical College of Georgia, Augusta University, 1120 15th Street, Augusta, GA 30912 USA.

**Keywords:** Photobiomodulation, TgF344 rats, Microglia recruitment, Mitochondria, Neuronal hemoglobin

## Abstract

Alzheimer's disease (AD) is the most common form of dementia in the elderly, causing neuronal degeneration and cognitive deficits that significantly impair independence and quality of life for those affected and their families. Though AD is a major neurodegenerative disease with vast avenues of investigation, there is no effective treatment to cure AD or slow disease progression. The present work evaluated the therapeutic effect of long-term photobiomodulation (PBM) treatment with continuous-wave low-level laser on AD and its underlying mechanism.

**Methods:** PBM was implemented for 2 min, 3 times per week for 16 months in 2-month-old transgenic AD rats. A battery of behavioral tests was performed to measure the effect of PBM treatment on cognitive dysfunction in AD rats. The effects of PBM therapy on typical AD pathologies, including amyloid plaques, intracellular neurofibrillary tangles, neuronal loss, neuronal injury, neuronal apoptosis, and neurodegeneration, were then assessed. The underlying mechanisms were measured using immunofluorescence staining, western blotting analysis, mass spectrometry, primary cortical and hippocampal cell cultures, and related assay kits.

**Results:** PBM treatment significantly improved the typical AD pathologies of memory loss, amyloid plaques, tau hyperphosphorylation, neuronal degeneration, spine damage, and synaptic loss. PBM treatment had several mechanistic effects which may explain these beneficial effects, including 1) regulation of glial cell polarization and inhibition of neuroinflammation, 2) preservation of mitochondrial dynamics by regulating fission and fusion proteins, and 3) suppression of oxidative damage to DNA, proteins, and lipids. Furthermore, PBM enhanced recruitment of microglia surrounding amyloid plaques by improving the expression of microglial IL-3Rα and astrocytic IL-3, which implies a potential role of PBM in improving Aβ clearance. Finally, our results implicate neuronal hemoglobin in mediating the neuroprotective effect of PBM, as Hbα knockdown abolished the neuroprotective effect of PBM treatment.

**Conclusion:** Collectively, our data supports the potential use of PBM treatment to prevent or slow the progression of AD and provides new insights into the molecular mechanisms of PBM therapy.

## Introduction

Alzheimer's disease (AD) is the most common form of dementia in the elderly with over six million individuals estimated to have AD in the US 1. AD is characterized by significant cognitive decline that can impair the ability to carry out daily activities, resulting in a reduced quality of life 2, 3. The major pathological hallmarks of AD include the development of extracellular β-amyloid plaques (via Aβ1-42 peptides) as well as intracellular neurofibrillary tangles (NFT) within the neuronal processes 2, 3. Due to these hallmarks, the vast majority of clinical research for the treatment of AD has been based on the hypothesis that an excess of cerebral Aβ accumulation can cause synaptic dysfunction and neuronal degeneration in the AD brain - especially in the hippocampus, the region at the core of the cortical learning and memory system 4, 5. Although the underlying mechanisms are unclear, AD progression and pathogenesis is widely accepted to be associated with a series of events initiated by neurotoxic levels of Aβ that lead to intracellular NFT formation, oxidative stress, neuroinflammation, and mitochondrial dysfunction leading ultimately to irreversible neuronal death 6-9. However, therapeutic strategies targeting the high levels of amyloid in the AD brain, which comprises the bulk of experimental AD drug development, have been largely unsuccessful 10, 11.

Several reasons have been proposed to explain the failure of clinical trials on AD 10, 12. First, AD is a multi-factorial and complex brain disease thought to involve several different mechanisms, although the precise molecular mechanisms are still unclear 10, 13. Based on this “lock-and-key” model, drug discovery in AD has primarily focused on a single target (e.g., Aβ or tau) 14. Therefore, clinical trials or animal studies with only a single target may not be effective in slowing down or reversing the progression of AD 13. Second, many studies have used transgenic mouse models that have early onset of the disease but don't display all the hallmarks of AD 15. Third, use of an inappropriate therapeutic time window in AD treatment may also contribute to the failure of treatments to successfully translate in the clinic 15. Recently, in recognition of this, more investigators have begun to focus on AD prevention or interventions at the early stage of AD rather than treatment at later stages 3, 16, 17.

Our group has been interested in developing non-invasive treatments for neurodegenerative diseases such as AD, since these could be easily and widely used if successful. In particular, we have recently focused on photobiomodulation (PBM) as one such potential therapy. PBM involves the use of relatively low levels of visible or near-infrared light (wavelength between ~600-1270 nm) to stimulate healing, relieve pain and inflammation, and preserve tissue function 18. As a non-invasive procedure, PBM treatment is practiced by directly applying low-energy laser to a specific area of interest on the body 19. The most well-understood mechanism underlying the beneficial mitochondrial effects of PBM treatment is that cytochrome C oxidase (CCO), complex IV in the mitochondrial respiratory chain, absorbs near-infrared photons. This leads to the displacement of inhibitory nitric oxide, increased mitochondrial membrane potential, and subsequently boosts ATP generation 6. However, the precise mechanism(s) of PBM modulation for the treatment of AD remain elusive 20. Traumatic brain injury, stroke, neurodegenerative diseases (AD and Parkinson's), and psychiatric disorders (depression, anxiety, insomnia, post-traumatic stress disorder (PTSD)) all respond well to PBM 20-23. Therefore, using a novel AD-like transgenic rat model, the TgF344-AD rat, the current study was designed to investigate the effects and underlying mechanism(s) of long-term PBM initiated at the early stages of AD. The TgF344-AD rat model was chosen because it closely models pathology observed in human AD, including strong age-related aβ accumulation with development of amyloid plaques, tauopathy with structures that resemble NFTs, neuronal loss, and age-related synaptic and cognitive defects 24.

## Methods

### Animals and experimental design

**Figure 1:**
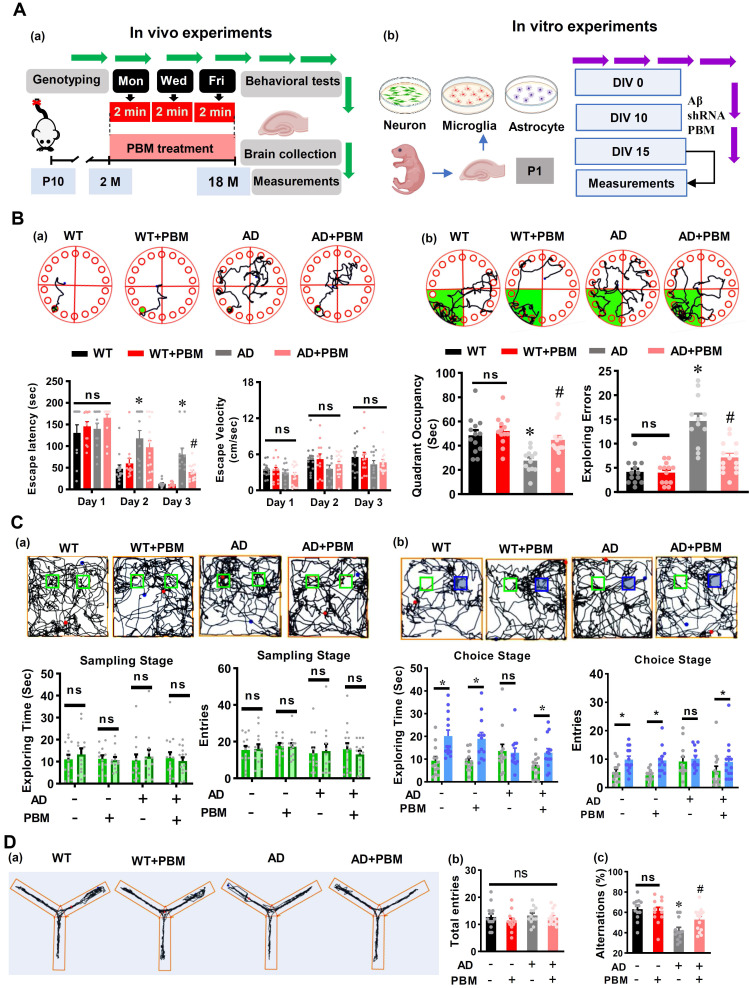
** PBM treatment attenuates cognitive deficits in AD-like rats. (A)** Schematic diagram of the experimental design for the *in vivo* study (a) and *in vitro* study. **(B)** Representative tracking plots of animals, escape latency, and escape velocity on the training day (a) and tracking plots, quadrant occupancy, and exploring errors on the probe trials in the Barnes maze test (b) were recorded and analyzed. **(C)** Representative tracking plots of rats, exploring time spent on two identical objects or on the original object (green) and novel object (blue), entries to the area where the objects located on sampling stage (a) and choice stage were recorded and analyzed. **(D)** The representative exploration tracks during Y maze (a), and analysis on total entries (b) and alternations (c). Data in behavioral tests are presented as mean ± SEM (n = 12-15). *P < 0.05 versus WT group, ^#^ *P* < 0.05 versus AD group. ns indicates no significant difference (*P* > 0.05).

Two-month-old male TgF344-AD rats and age-matched wild-type Fischer 344 were used in our study. The Tg344-AD rats (RRRC, NIH) 24 were divided into the following groups after genotyping: (1) WT animals with Sham laser treatment (WT, n = 16), (2) WT rats with PBM treatment (WT+PBM, n = 16), (3) AD rats with Sham laser treatment (AD, n = 16) and (4) AD rats with PBM treatment (AD+PBM, n=16). The animals in WT and AD groups have the same restraint as in PBM-treated groups. The only difference between animals with or without PBM treatment is whether we turned on the laser. Genotyping was performed to distinguish AD and wild-type animals in the offspring as described in our previous study 2. In brief, a portion of the rat's distal tail (~3 mm) was removed using a sterile sharp scalpel. The tail tips were then placed into a tissue collection tube and incubated with extraction solution and tissue preparation solution for 10 min at room temperature and then boiled for another 3 min. The neutralization solution, PCR master mix, and primers were then mixed with the sample. The following primers were used for the genotyping: R699 APP forward sequence: 5'-CCGAGATCTCTGAAGTGAAGATGGATG-3', R699 mPrp forward: 5'-CCTCTTTGTGACTATGTGGACTGATGTCGG-3' and R699 mPrp reverse: sequence: 5'-GTGGATACCCCCTCCCCCAGCCTAGACC-3'. The genotyping process was repeated two times for each rat. Representative result for AD rats is shown in **Figure S1**. As shown in **Figure 1A(a),** the 2-min PBM treatment was initiated at 2 months of age, given 3 times/week (Monday, Wednesday, and Friday) and continued until the animals were 18 months old. Behavioral tests were performed at 18 months old followed by brain collection and measurements. All procedures were approved by the Institutional Animal Care and Use Committee (IACUC) of Augusta University and were performed in compliance with National Institutes of Health guidelines.

### PBM treatment

The non-contact transcranial PBM treatment with 808 nm continuous-wave low-level laser (MDL-III-LED, model 808M100, Dragon Lasers) was performed as described in our previous studies 25, 26. For the *in vivo* studies, 2-min daily PBM treatment was initiated at 2 months of age, given 3 times/week (Monday, Wednesday, and Friday) and continued until the animals were 18 months old. All animals were shaved before PBM treatment. During PBM treatment, animals were restrained in a transparent plastic cone (**Figure S2A**), and the eyes were covered with aluminum foil to avoid interference from the visible guide light. The restraint did not induce anxious-depressive behavior (**Figure S3A-E**). The irradiance received on the scalp and cortex was 350 mW/cm^2^ (fluence: 42 J/cm^2^) and 25 mW/cm^2^ (fluence: 3 J/cm^2^), respectively. The distance between the laser tip and the animal's scalp was adjusted to 35 cm to generate a 1.5 cm^2^ laser spot on the animal's scalp (Beam divergence, full angle: < 3.0 mrad). The device information for the animal study is shown in **Figure S2B.** For the PBM treatment on the cell, the laser beam spot was adjusted to cover the cell culture well, and the irradiance received by the cell was adjusted to 25 mW/cm^2^.

### Cell culture and *in vitro* experimental design

As illustrated in **Figure 1A(b)**, the primary cortical and hippocampal cell cultures were prepared from postnatal day 1 (P1) Sprague-Dawley rats. For neuronal culture, in brief, the dissociated neurons were plated on poly-D-lysine (PDL, 50 µg/mL, Thermo Fisher) coated wells in Neurobasal medium supplemented with L-glutamine (Thermo Fisher), B27 (Thermo Fisher), and penicillin-streptomycin (Thermo Fisher). The reversed (Aβ42‐1), Aβ1-42 (Sigma-Aldrich), scrambled-shRNA (Vector Biolabs), and Hbα-A2-shRNA (Vector Biolabs) were added to the cell culture medium at *in vitro* day 10 (DIV 10). The primary microglia and astrocyte cultures were performed as described previously 27. The dissociated cell suspension was cultured on PDL-coated flasks with Dulbecco's modified Eagle medium (Thermo Fisher) supplemented with 10% fetal bovine serum (FBS, Thermo Fisher). On DIV8, astrocytes were at the bottom of the cell culture dishes with microglia on top. To collect microglia, the dishes were placed on a shaker inside a humidified 37 °C, 5% CO2 incubator, and shaken for 2 h at 180 rpm. The microglia were collected in the supernatant. Thereafter, the fresh cell culture medium was added and continue shaking at 240 rpm for another 6 h to remove the oligodendrocyte precursor cells in the supernatant and added trypsin-EDTA (Thermo Fisher) to allow astrocyte detachment from the culture flask. The culture medium with microglia or astrocyte was then spun down and plated on appropriate cell culture plates. PBM treatment was carried out at DIV10, and the cells were fixed, stained, or measured on DIV15. The following HBA-A2-shRNA were used to target Hbα: 5'- CACC GGACAAATTCCTTGCCTCTGTCTCGAGACAGAGGCAAGGAATTTGTCC TTTTT-3'.

### Barnes maze task

The Barnes maze task was performed to measure hippocampus-dependent spatial learning and memory following our previously published procedures 28. The Barnes maze used in our study consists of a circular platform (100 cm high) with 18 holes around the circumference. The platform was divided into four quadrants including a target quadrant with a hidden box under the escape hole. Briefly, the task was divided into 3 training trials and a probe trial. During the 3-min training on the first three days, the animals were placed on the center of the platform and allowed to explore the platform freely. During the training trials, if the animals could not find the target box within 3 min, the researcher gently led the animal to the hidden box. The traces of the rats and the time spent finding the escape hole were recorded by ANY-maze video tracking software (Stoelting, Wood Dale, IL, USA) with an overhead video camera. On the probe day, the hidden box was removed and the escape hole was blocked. The animals were allowed to explore the platform for 90 s. The total time spent in the target quadrant and the number of exploring errors were recorded on the probe trials.

### Novel object recognition test

Novel Object Recognition tests were performed to measure recognition memory as described previously by our laboratory 29. The test was divided into two sessions. During the sampling stage, the animals were presented with identical objects and allowed to explore the box ((height: 40 cm; width: 50 cm; length: 50 cm) for 5 min. On the second day (choice stage), one of the objects was replaced by a novel object, and the animals were allowed to explore the box for another 5 min. The time spent exploring each object and the traces of each animal were recorded using ANY-maze video tracking software.

### Y-Maze spontaneous alternation test

The Y-Maze spontaneous alternation test was adopted to assess short-term spatial working memory as described in our previous study 21. The Y-maze apparatus used in our study consisted of three arms joined in the middle for a “Y” shape. The Y-maze test is based on the spontaneous natural behavior of rats to explore the new arms rather than returning to previously visited arms. In our study, the rats were allowed to explore the maze for 5 min, recording the number of total arm entries and spontaneous alternations, defined as consecutive entries in 3 different arms. The percentage of spontaneous alternation was calculated with the following formula: Spontaneous alternation% = (# of spontaneous alternations/ (total arm entries-2) × 100%.

### Sucrose preference test

The sucrose preference test was adopted to test depressive-like behavior 17. In the first two days, the rats were given two bottles of plain water, followed by the sucrose solution (2%) for another 2 days. Following the adaptive phase, the rats underwent 2 days of water deprivation, followed by free choice of plain water or sucrose solution for 8 h. Sucrose preference was calculated according to the following formula: Sucrose preference = (volume of sucrose solution consumed)/(total volume of plain water and sucrose intake) × 100%.

### Forced swim test

The forced swim test was performed to assay depressive-like behavior as described in our previous study 2, 17. Briefly, the animals were placed into a transparent tank filled with water at room temperature (24 ± 1 °C) for 6 min. The immobile time was recorded using ANY-maze video tracking software.

### Tail suspension test

The tail suspension test was conducted to detect anxious-depressive-like behavior following the description in our previous study 17. In brief, the rats were suspended in the middle of the suspension box 50 cm above the ground by their tails. The immobile time was recorded during the 6 min of testing.

### Open field test

The open field test was adopted to measure anxiety-like behavior as described previously 17. A square black box was used for the assessment. In brief, the rat was placed in the arena and allowed to freely move in the box for 5 min. The time spent in the central zone and the number of defecations left in the box were recorded using ANY-maze video tracking software. 70% ethanol was used to clean the box between each test.

### Elevated plus maze

The elevated plus-maze was applied to assess anxiety-related behavior as described by previous studies 17, 21. The apparatus elevated plus-maze was 50 cm above the floor with two oppositely positioned open arms, two oppositely positioned closed arms, and a central zone. The two oppositely positioned closed arms were surrounded by 50-cm-high walls. The animals were placed at the central zone and allowed to freely explore the maze for 5 min. Open arms entries and the amount of time remained in the open arms were recorded using ANY-maze video tracking software.

### Brain collection and tissue preparation

Brain collection was performed after behavioral tests as described in our previous study 29. In brief, the rats were sacrificed and the brain was quickly collected following deep isoflurane anesthesia. One side of the brain was post-fixed with 4% paraformaldehyde (Thermo Fisher) for the tissue section, and the remaining hemisphere was micro-dissected to get cortex and hippocampus. The brain section (25 µm) was prepared using a Leica Rm2155 microtome and stored in stock solution (FD NeuroTechnologies, Inc., Columbia, MD, USA) for immunofluorescence staining. The protein from both the cortex and hippocampus were prepared using a motor-driven Teflon homogenizer with a 400 μL ice-cold mixture of homogenization buffer, protease, and phosphatase inhibitors. The homogenates were centrifuged with appropriate speed and time to get either the total protein or subcellular fractions as described in our previous study 26.

### Immunofluorescence staining

Immunofluorescence staining was performed as described in detail previously by our laboratory 29, 30. In brief, coronal brain slices were incubated with 0.4% Triton X-100 (Thermo Fisher) for 5 h followed by 10% normal donkey serum for another 1 h at room temperature. Next, the brain slices were incubated with appropriate primary antibodies in 0.1% Triton X-100 overnight at 4 °C. The following antibodies were used in our study: anti-MBP (Proteintech), MAP2 (Thermo Fisher), NeuN (Proteintech), spinophilin (Abcam), synaptophysin (Abcam), 4G8 (Covance), PHF1 (Abcam), Iba-1 (Proteintech), IL-3Rα (Abcam), IL-3 (Abcam), GFAP (Thermo Fisher), CD206 (Proteintech), CD16/32 (Proteintech), C3d (Thermo Fisher), S100A10 (Thermo Fisher), Tom20 (Proteintech), 8-OHdG (Abcam), 4-HNE (Abcam), Hbα (Abcam), Hbβ (Abcam), Cle-caspase 3 (Cell Signaling Technology), Mfn2 (Abcam). After the incubation with the primary antibody, brain slices were washed four times with 0.1% Triton X-100 and then incubated with appropriate fluorescent-labeled secondary antibodies (594/647/488, Thermo Fisher Scientific) for 1 h at room temperature. After incubation with secondary antibodies, the brain slices were then washed and mounted with DAPI Fluoromount-G® Mounting Medium (SouthernBiotech, Birmingham, AL, USA). The LSM700 Meta confocal laser scanning microscope (Carl Zeiss, White Plains, NY, USA) was adopted to capture fluorescent images. All fluorescent images were analyzed using ImageJ software (Version 1.49, NIH, Bethesda, MD, USA) or Imaris software (Bitplane AG, Zürich, Switzerland). 3D reconstruction was performed using Imaris software following the instructions described in previous studies 28, 31. The smoothing was set at 0.4 μm for all images and channels. An appropriate threshold was applied to remove the background noise and non-specific signals. For astrocytic volume analysis, a cell volume over 200 μm^3^ was selected for further analysis.

### Western blotting analysis

Western Blotting Analysis was performed following the protocol described in a previous study 32. In brief, protein (30-50 μg/lane) was separated by sodium dodecyl sulfate-polyacrylamide gel electrophoresis (SDS-PAGE) and then transferred onto a polyvinylidene difluoride (PVDF) membrane. After incubation with 3% bovine serum albumin (BSA) for 30 min, the membranes were then incubated at 4 °C overnight with the following antibodies: Bax (Abcam), PSD95 (Thermo Fisher), spinophilin (Abcam), synaptophysin (Abcam), GAPDH (Abcam), CD86 (Proteintech), iNOS (Abcam), CD32 (Proteintech), TGFβ (Proteintech), ARG (Proteintech), MFF (Proteintech), FIS1 (Proteintech), Drp1 (BD Biosciences), MFN2 (Proteintech), OPA1 (BD Biosciences), COX4 (Proteintech), Hbα (Abcam), Bcl-2 (Santa Cruz Biotechnology), and β-actin (Proteintech). After three washes, the membranes were then incubated with HRP-conjugated secondary antibody (Cell Signaling) at room temperature for 1 h. After three washes, the HRP chemiluminescent substrate was added to completely cover the surface of the membranes. A cold CCD digital imaging system was applied to capture the images, and the ImageJ software (Version 1.49, NIH) was used to analyze the intensity of the band.

### Measurement of Aβ levels

Aβ1-42 or Aβ1-40 ELISA kits (Invitrogen) were used to measure Aβ species. Detergent-soluble Aβ40 or Aβ42 were measured following the manufacturer's instructions and as described in a previous study 33. For the insoluble Aβ1-42 or Aβ1-40, the protein samples were pre-treated with 5 M guanidine-HCl (pH 8.0) buffer before ELISA measurement.

### TUNEL assay and F-Jade C staining

The TUNEL assay and F-Jade staining were performed to detect cellular apoptosis and degenerating neurons, respectively 34. A Click-iT® Plus TUNEL assay kit (Thermo Fisher Scientific) was adopted to label cellular apoptosis following the manufacturer's instructions. For the F-Jade C Staining, the brain slices were incubated with Fluoro-Jade C working solution (Sigma-Aldrich, St. Louis, MO, USA) in the dark for 20 min at room temperature following the manufacturer's protocol. The brain slices were then rinsed 3 times with distilled water and coverslipped with a mounting medium. Image capture and analysis was performed the same as immunofluorescence staining.

### Caspase 3 and caspase 9 activity

The activity of caspase 3 and caspase 9 was measured using fluorometric substrates as described in our previous studies 28, 35. In brief, proteins from the cortex and hippocampus were incubated with the fluorometric substrates Ac-DEVD-AMC (caspase 3 substrate) and Ac-LEHD-AMC (caspase 9 substrate) at 37 °C for 1 h. Thereafter, the mixture of the protein and the substrate was measured on Synergy HT Microplate reader (BioTek Instruments Inc, Winooski, VT, USA) with excitation at 360 nm excitation/460 nm emission. The optical density value (OD) was recorded and presented as a percentage of change compared with WT animals.

### Hemoglobin colorimetric assay kit

A hemoglobin colorimetric assay kit (Cayman Chemical, Ann Arbor, MI, USA; Item No. 700540) was used to measure hemoglobin following the manufacturer's instructions. In brief, 20 μL sample was mixed with 180 μL of hemoglobin detector followed by incubation at room temperature for 15 min. The standard wells were filled with 200 μL of hemoglobin standard. The standard and the sample wells were read at 560 nm.

### Electron microscopy analysis

The fresh brains of rats were quickly removed and post-fixed at 4 °C in 2% paraformaldehyde and 2% glutaraldehyde for 2 h. The collected brains were then rinsed 3 times with phosphate buffer followed by post-fixation in 1% osmium tetroxide for another 2 h. After three washes in phosphate buffer and dehydration in ethanol and propylene oxide series, the brains were embedded in EmBed812. 70-nm ultrathin sections were cut using a Leica ultramicrotome and counterstained with lead citrate and uranyl acetate. A JEM 1230 transmission electron microscope (JEOL USA Inc., Peabody, MA) with an UltraScan 4000 CCD camera was applied to capture the images.

### Spine density analysis

Golgi staining was adopted to determine the changes of spines according to the manufacturer's instructions (FD Rapid GolgiStain TM kit, FD NeuroTechnologies, Inc). Briefly, the fresh rat brains were incubated with pre-prepared impregnation solution at room temperature for 2 weeks in the dark. Thereafter, the collected brains were then incubated with manufacturer-provided solution C for another 96 h at room temperature. The brain sections of 200 μm were then prepared and mounted with solution C on gelatin-coated slides and allowed to dry naturally for 2 days. Thereafter, the brain sections were then rinsed and incubated with a mixture of manufacturer-provided solutions D and E for 10 min. The brain sections were subsequently rinsed, dehydrated, and cleared with xylene and ethanol. The images were captured using an OLYMPUS IX70 microscope. The ImageJ software was used to process and analyze the images.

### Inflammatory cytokines assay

The Indirect Enzyme-Linked-Immunosorbent-Assay (ELISA) was applied to measure inflammatory cytokines as described in previous studies 3, 17. In brief, the samples with the same amount of protein were loaded into the polyvinyl chloride ELISA microplate (Corning) overnight at 4 °C. After 3 washes, the blocking buffer was loaded into the microplate for another 1 h. After removing the blocking buffer, the following antibodies were added: TNF-α, NFκB, IL-1β, IL-4, IL-10, and IL-13, and incubated at room temperature for 1 h. Afterward, TMB development solution was added and incubated for 30 min. After adding the stop solution, the microplate was read at 450 nm using a spectrophotometer (Bio-Rad; Hercules, CA, USA). All data were calculated and expressed as percent changes versus the WT group.

### Total antioxidant capacity assay

Total antioxidant capacity assay (Cayman Chemical, Ann Arbor, MI, USA) was applied to assess the total antioxidant capacity as described in our previous studies 28. Briefly, equal amounts of total protein from each sample were incubated with metmyoglobin (10 µL) and chromogen (150 µL) following the instruction of the vendor. Afterward, 40 µL hydrogen peroxide working solution was added to the plates and incubated for 5 min on a shaker. The plates were then measured using a spectrophotometer at 750 nm. The OD value was recorded and analyzed using a Trolox standard curve. The data was expressed as a percentage of the WT group.

### Lipid peroxidation (MDA) assay

The levels of malondialdehyde in the cortex and hippocampus were determined using a lipid peroxidation (MDA) assay kit (ab118970; Abcam, Cambridge, United Kingdom). Briefly, 200 µL of protein samples with equal amount proteins were incubated with 600 µL of manufacturer-provided TBA reagent at 95 °C for 60 min. After cooling in an ice bath for 10 min, the supernatant was transferred to a 96-well microplate and measured using a microplate reader at 532 nm. The OD value was recorded and analyzed.

### Protein carbonylation determination

An OxiselectTM protein carbonyl ELISA Kit (Cell Biolabs Inc, San Diego, CA, USA) was adopted to determine protein carbonylation in both the cortex and hippocampus following the instructions of the vendor. In brief, 100 μL protein samples were added onto the 96-well protein plate provided by the vendor and incubated overnight at 4 °C. After 3 rinses, the dinitrophenylhydrazine (DNPH) working solution (100 μL) was mixed with the protein samples for 45 min at room temperature. Afterward, 100 μL of anti-DNP was added and incubated at room temperature for 1 h. After another 3 washes, the HRP-conjugated secondary antibody was added to the plate and incubated for 1 h. Thereafter, substrate solution was added and incubated for 10 min at room temperature. Finally, a microplate reader was applied to measure the samples.

### Mass spectrometry

Mass spectrometry was performed as described in our previous study 21. The mass spectrometry analysis was performed by our proteomic core facilities. In brief, the prepared proteins were precipitated using trichloroacetic acid followed by digestion using trypsin. After digestion, the peptide was subjected to LC-MS/MS analysis as described in a previous study 36. The Z score was calculated for further analysis.

### JC-1 staining

The JC-1 staining was used to measure mitochondrial membrane potential according to the instructions of the vendor. In brief, cells were incubated with JC-1 (2.0 µg/mL) at 37 °C for 20 min. After 3 washes with PBS, the cells were mounted and captured using LSM700 Meta confocal laser scanning microscope (Carl Zeiss, White Plains, NY, USA).

### Detection of hypoxic cells

Hypoxic neuronal cells were detected using a Hypoxyprobe-1 Kit (Hypoxyprobe, Burlington, MA) following the manufacturer's instructions and the descriptions in a previous study 37. In brief, the neurons were incubated with pimonidazole hydrochloride (200 mmol/L; Hypoxyprobe-1, Chemicon)) for 4 h. After 3 washes, the cells were then incubated with monoclonal anti-pimonidazole antibody followed by incubation with Alexa Fluor anti-mouse secondary antibodies. The images were taken using The LSM700 Meta confocal laser scanning microscope (Carl Zeiss, White Plains, NY, USA).

### Neuronal viability measurement

Neuronal viability was assessed at DIV 16 using fluorescein diacetate (FD, Sigma) and propidium iodide (PI, Sigma) staining, and 2,5-diphenyl-2H-tetrazolium bromide (MTT) assay. For FD/PI staining, cells were incubated with FD (10 μg/mL) and PI (5 μg/mL) in the dark for 20 min. After washes, a confocal laser microscope was used to capture the images. The MTT assay was also applied to test neuronal viability 38. Briefly, neurons were cultured in 96-well plates and incubated with MTT labeling reagent (final concentration 0.5 mg/ml) for 4 h. After washes, dimethyl sulfoxide (DMSO, 150 μL/well) was added to each well, and the plate was place on a plate shaker at room temperature for 10 min. The optical density was measured using a spectrophotometer at 570 nm.

### Statistical analysis

SigmaStat software (Systat Software; San Jose, CA, USA) was applied to perform statistical analyses. One-way analysis of variances (ANOVAs) with Student-Newman-Keuls (S-N-K) post hoc tests were used to perform the statistical comparisons between groups. Two-way ANOVA were applied to analyze all dependent variables with multiple time points (group*time). If a significant group*time interaction occurred, Student-Newman-Keuls method post hoc tests were applied to analyze the difference between groups at each time point. Comparisons between two groups were performed using the Student's t-test (two-tailed). All data were expressed as means ± SEM. *P* < 0.05 was considered statistically significant.

## Results

### PBM treatment attenuates cognitive dysfunction in AD-like rats

To test the effect of PBM treatment on cognitive dysfunction in AD rats, the Barnes maze task and novel object recognition test were performed at 18 months of age. As shown in **Figure 1B**, rats were subjected to training trials on the first 3 days. On day 2, AD rats spent a significantly longer time finding the escape box compared with WT rats (*P* = 0.0018), and there was no difference between AD rats and AD rats with PBM treatment (**Figure 1B(a),** *P* = 0.162). However, AD rats with PBM treatment spent significantly reduced time in finding the escape box on day 3 (*P* = 0.00751), and there was no difference in the escape velocity between groups (*P* > 0.05), indicating the differences between groups in escape latency was not due to the differences in escape velocity (**Figure 1B(a)**). In addition, during the probe trials, AD rats spent significantly less time in the target quadrant (*P* = 0.00456) and showed more exploring errors on probe day than WT rats (*P* < 0.001), which was alleviated by PBM treatment (**Figure 1B(b)**). To evaluate recognition memory, novel object recognition tests were performed. As shown in **Figure 1C(a)**, during the sampling stage, rats did not show any preferences in exploring two identical objects in all groups (*P* > 0.05), as evidenced by exploring time and entries to the zone of objects. However, on the choice stage (**Figure 1C(b)**), WT rats and PBM-treated AD animals showed an increased preference in exploring the novel object (blue) compared with AD rats. Next, the Y maze test was performed to measure the short-term working memory. As shown in **Figure 1D**, although animals in all groups present a similar arm entry **(Figure 1D(b),** *P* > 0.05**)**, AD rats had a significantly reduced alternation compared with WT animals (*P* < 0.001) and PBM-treated AD animals (*P* = 0.0224) **(Figure 1D(c))**, indicating PBM treatment alleviated the impairment of short-term working memory in AD rats. No significant differences between WT animals with and without PBM treatment were detected (*P* > 0.05).

### PBM treatment ameliorates neuronal injury, neuronal apoptosis, and degeneration

**Figure 2:**
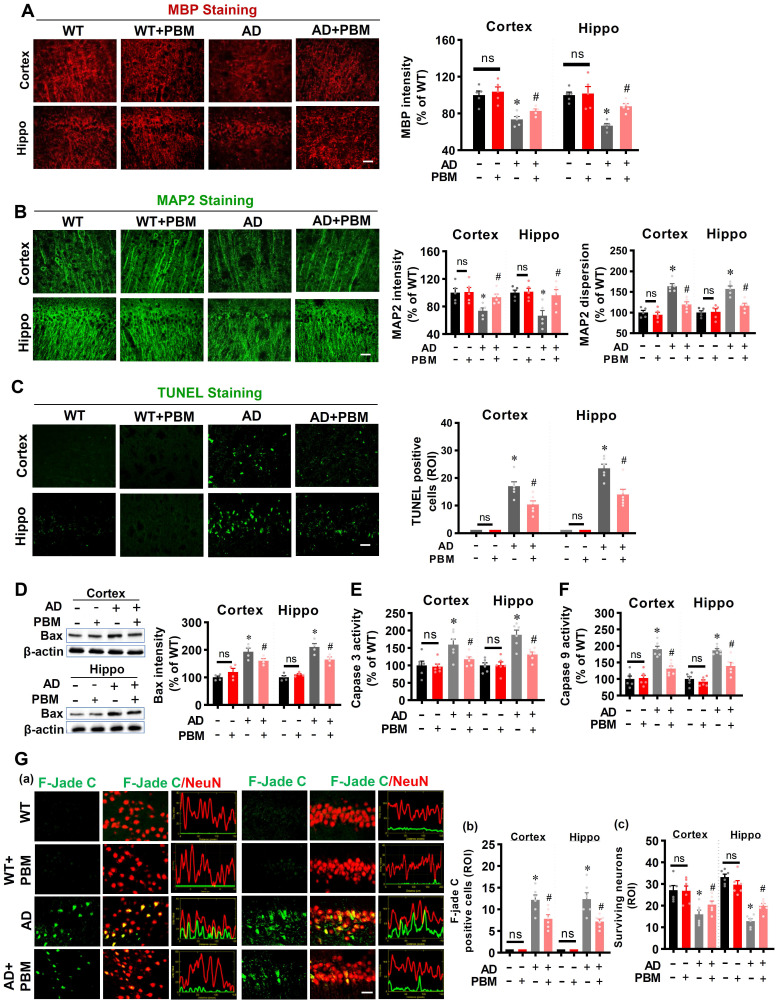
** PBM treatment ameliorates neuronal injury, neuronal apoptosis, and neuronal degeneration. (A)** Representative images of MBP staining and the relative fluorescence intensity compared with the WT group in both the cortex and hippocampus (Hippo). **(B)** Representative images of MAP2 staining and the relative fluorescence intensity. **(C)** TUNEL staining was performed to analyze cellular apoptosis.** (D)** Western blot analysis of Bax protein expression in both the cortex and hippocampus. **(E)** Caspase-3 activity was determined using a caspase-3 activity kit and expressed as a percentage of change compared with the WT group. **(F)** Caspase-9 activity was determined using a caspase-9 activity kit. **(G)** Typical staining of F-Jade C and NeuN in both the cortex and hippocampus. Line-scan analysis showed a strong co-localization of NeuN and F-Jade C signals in the AD group (a). Quantitative analyses of F-Jade C positive cells (b) and surviving neurons (c) in both the cortex and hippocampus. Scale bar = 20 µm. Data are presented as mean ± SEM (n = 4-6). *P < 0.05 versus WT group, ^#^ *P* < 0.05 versus AD group. ns indicates no significant difference (*P* > 0.05).

Next, to investigate the effects of PBM on neuronal injury, MAP2 and MBP were performed to measure neuronal injury. As shown in **Figure 2A**, MBP staining showed AD rats had significantly decreased MBP intensity in both the cortex (*P* = 0.00136) and hippocampus (*P* < 0.001), which was significantly reversed in PBM-treated AD rats (Cortex: *P* = 0.0443; Hippocampus: *P* = 0.0005). Furthermore, as shown in **Figure 2B**, MAP2, an early and sensitive marker for neuronal damage, presented significantly decreased fluorescent intensity and increased dispersion in the AD animals compared to WT (Intensity-cortex: *P* = 0.00826; Intensity-Hippocampus: *P* = 0.04826; Dispersion-cortex: *P* < 0.001; Dispersion-Hippocampus: P = 0.00013) and PMB-treated AD animals (Intensity-cortex: *P* = 0.0138; Intensity-Hippocampus: *P* = 0.0297; Dispersion-cortex: *P* = 0.0016; Dispersion-Hippocampus: *P* = 0.0023), suggesting PBM treatment significantly alleviated neuronal injury in AD rats. Furthermore, the effect of PBM on cellular apoptosis was measured using TUNEL staining. As shown in **Figure 2C**, the number of TUNEL-positive cells was significantly increased in both the cortex and hippocampus of AD animals compared with WT animals (Cortex: *P* < 0.001; Hippocampus: *P* < 0.001) and PBM-treated AD rats (Cortex: *P* = 0.011; Hippocampus: *P* = 0.0034), suggesting cellular apoptosis in the AD animals was alleviated by PBM treatment. Consistent with these results, as shown in **Figure 2D-F**, the activity of caspase 3, caspase 9 and the level of Bax in the cortex and hippocampus of AD rats were significantly increased compared with the WT rats and PBM-treated AD rats, supporting the anti-apoptotic efficacy of PBM in AD rats. Subsequently, Fluoro-Jade C staining was performed to measure neuronal degeneration. As shown in **Figure 2G**, cortex and hippocampus from AD rats displayed significantly increased F-Jade C positive cells (Cortex: *P* =0.0162; Hippocampus: *P* = 0.0101), demonstrating neuronal degeneration was significantly increased in AD animals. In contrast, PBM treatment significantly ameliorated neuronal degeneration.

### PBM treatment alleviates the damage to spine synapses and dendritic spines

**Figure 3:**
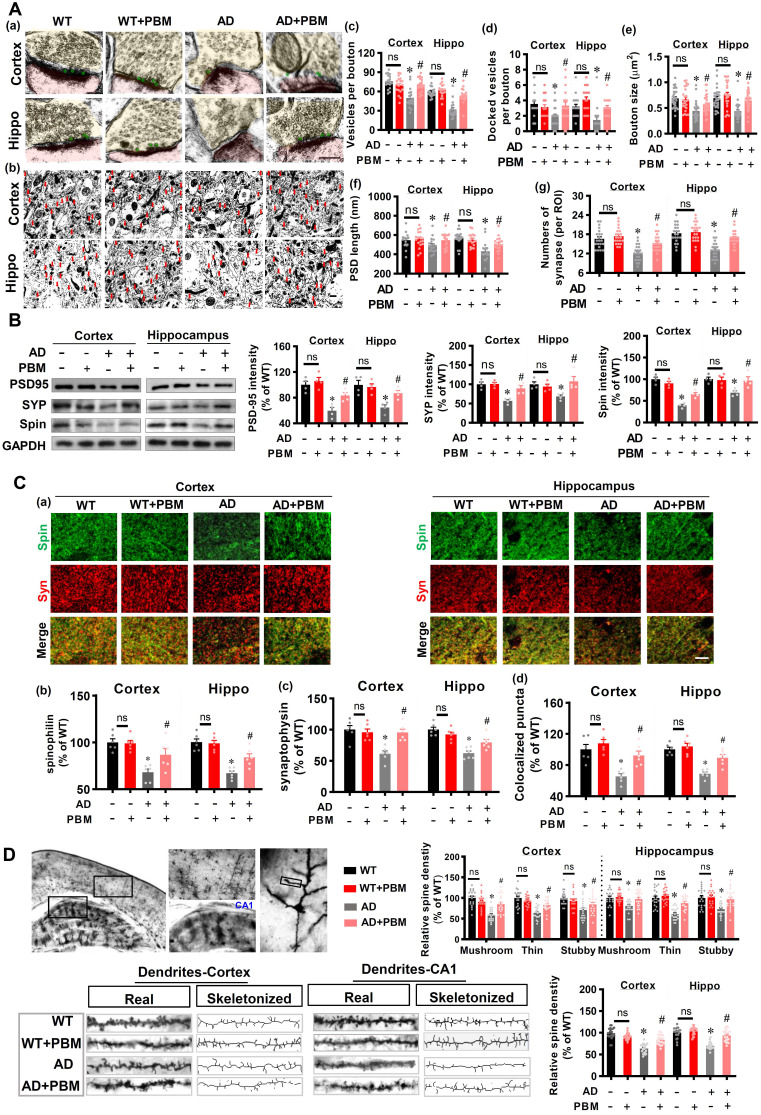
** PBM treatment alleviates the changes of synapse and dendritic spine density. (A)** Ultrastructural analysis of synapses using electron microscopy in both the cortex and hippocampus (a, Scale bar = 200 nm). Low-magnification photograph showing the numbers of synapses (red arrow) in the region of interest of cortex and hippocampus (b, Scale bar = 10 µm). The number of vesicles per bouton (c), docked vesicles (d), bouton size (e), PSD length (f), and the number of synapses (g) were analyzed. Data are presented as mean ± SEM (n = 18)**. (B)** Western blot analysis of synaptophysin (a presynaptic marker), spinophilin (a spine marker), and PSD-95 (a postsynaptic marker). Data are presented as mean ± SEM (n = 4). **(C)** Representative images of synaptophysin and spinophilin staining in both the cortex and hippocampus were shown in (a, Scale bar = 10 µm). The relative fluorescence intensity of spinophilin (b) and synaptophysin (c), and colocalized puncta between the two channels were qualified. Data are presented as mean ± SEM (n = 6)**. (D)** Representative images of dendritic segments stained by Golgi staining. The dendrite and spine morphologies in both the cortex and hippocampus were analyzed using Image J. Data are presented as mean ± SEM (n = 20). *P < 0.05 versus WT group, ^#^ *P* < 0.05 versus AD group. ns indicates no significant difference (*P* > 0.05).

Spine synapses and dendritic spine density are essential to learning and memory. Therefore, we analyzed the effect of PBM on spine synapses and dendritic spines. First, electron microscopic analysis was performed to investigate synaptic structure in both cortex and hippocampus. As shown in **Figure 3A,** the numbers of vesicles per bouton (Cortex: *P* < 0.001; Hippocampus: *P* < 0.001), docked vesicles (Cortex: *P* =0.00281; Hippocampus: *P* = 0.001097) at the presynaptic membrane, bouton size (Cortex: *P* < 0.001; Hippocampus: *P* < 0.001), PSD length (Cortex: *P* = 0.0489; Hippocampus: *P* < 0.001), and the numbers of synapses (Cortex: *P* < 0.001; Hippocampus: *P* < 0.001) in the cortex and hippocampus of AD animals were significantly decreased compared to WT animals. Intriguingly, PBM treatment significantly attenuated these changes in AD animals. Furthermore, expression of the presynaptic marker synaptophysin, the postsynaptic marker PSD95, and the dendritic spine marker spinophilin were measured. As shown in **Figure 3B**, AD animals displayed decreased expression of synaptophysin (Cortex: *P* = 0.0007; Hippocampus: *P* = 0.00641), PSD95 (*P* = 0.002; Hippocampus: *P* = 0.0053), and spinophilin (Cortex: *P* < 0.001; Hippocampus: *P* = 0.0025) in both the cortex and hippocampus compared with WT animals. In contrast, PBM treatment of AD animals ameliorates this decrease. Consistent with these results, immunostaining for synaptophysin and spinophilin displayed decreased levels (synaptophysin-cortex: *P* = 0.0007; synaptophysin-hippocampus: *P* < 0.001; spinophilin-cortex: *P* < 0.001; spinophilin-hippocampus: *P* < 0.001) in AD animals and was preserved in PBM-treated AD animals (synaptophysin-cortex: *P* = 0.0008; synaptophysin-hippocampus: *P* < 0.001; spinophilin-cortex: *P* = 0.026; spinophilin-hippocampus: *P* = 0.0042) (**Figure 3C**). Furthermore, the effect of PBM on spine morphology was measured using Golgi staining. As shown in **Figure 3D**, spine density in both the cortex and hippocampus were skeletonized and analyzed. The results revealed that the spine density was significantly decreased, as compared to WT animals (Cortex: *P* < 0.001; Hippocampus: *P* < 0.001). PBM treatment, however, significantly preserved the spine density of AD animals (Cortex: *P* < 0.001; Hippocampus: *P* < 0.001). Spines in the cortex and hippocampus were further classified into mushroom, thin and stubby. In AD groups, the density of the mushroom, thin and stubby spines were significantly reduced. In contrast, PBM treatment attenuated the changes of spine density.

### PBM treatment attenuates amyloid load and abnormal tau hyperphosphorylation

**Figure 4:**
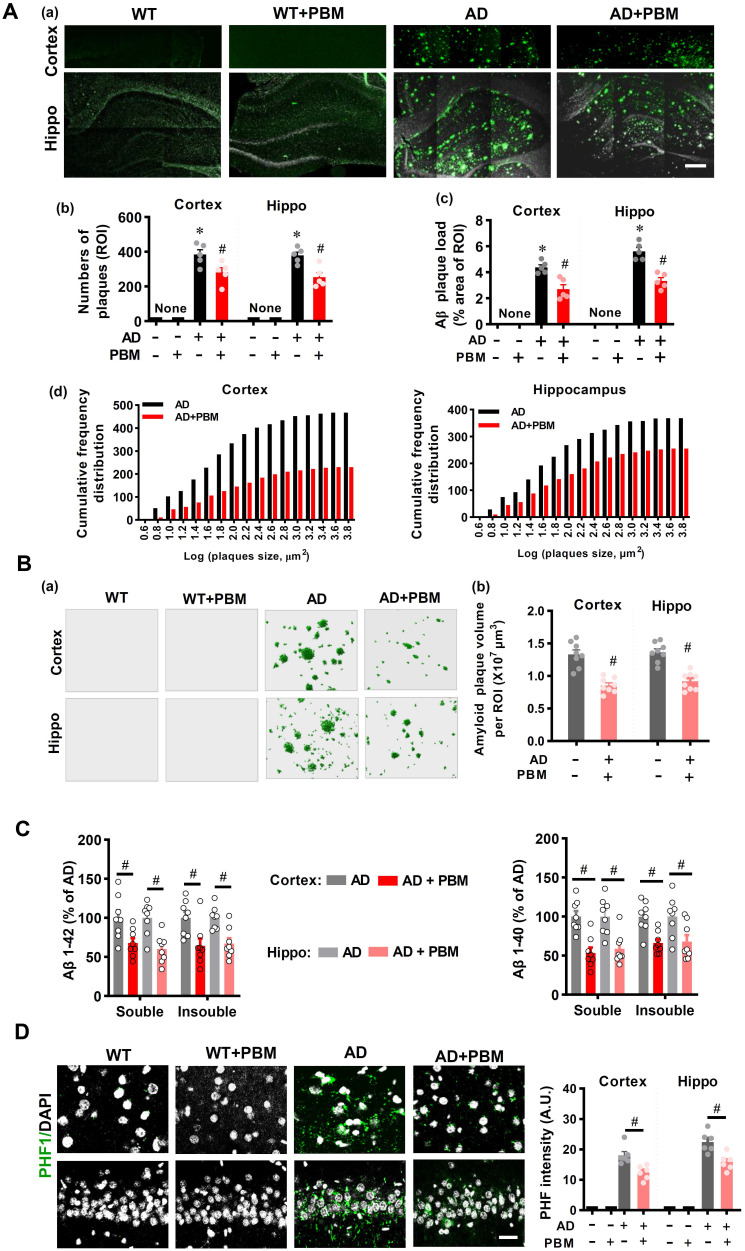
** PBM treatment attenuates amyloid load and abnormal Tau hyperphosphorylation. (A**) Representative immunofluorescence staining for Aβ (4G8) in both the cortex and hippocampus (a). The numbers of plaques (b), Aβ plaque load (c), and the cumulative frequency distribution of the size of the plaque (d) in both the cortex and hippocampus were analyzed. Scale bar = 400 µm. n = 5. **(B)** Representative 3D reconstruction of amyloid plaques (a) and the analysis of amyloid plaques volume (b). **(C)** Detergent-soluble and detergent-insoluble Aβ (1-40) and Aβ (1-42) were measured in both the cortex and hippocampus. **(E)** Representative immunofluorescence staining for PHF1. Scale bar = 20 µm. Data are presented as mean ± SEM (n = 6). *P < 0.05 versus WT group, ^#^ *P* < 0.05 versus AD group.

Extracellular Aβ accumulation-induced amyloid plaque and intracellular tau hyperphosphorylation-induced neurofibrillary tangles are considered hallmarks of AD. Therefore, we next analyzed whether PBM treatment affected amyloid load and tau hyperphosphorylation in the cortex and hippocampus of AD rats. As shown in **Figure 4A(a)**, AD rats at 18 months of age presented obvious amyloid plaques as compared with WT animals, which was alleviated by PBM treatment. Further analysis on the numbers of plaques and the plaque area showed that AD animals had significantly increased numbers of plaques and plaque area in both the cortex and hippocampus of AD rats as compared to WT animals (Number-cortex: *P* < 0.001; Number-hippocampus: *P* < 0.001; area-cortex: *P* < 0.001; area-hippocampus: *P* < 0.001) and PBM-treated AD animals (Number-cortex: *P* = 0.030; Number-hippocampus: *P* = 0.005; area-cortex: *P* = 0.003; area-hippocampus: *P* = 0.0004) (**Figure 4A (b and c)**). In addition, as shown in **Figure 4A (d) and Figure 4B**, the numbers of amyloid plaques of different sizes and the total volume of Aβ plaques were also decreased in the cortex and hippocampus of PBM-treated AD rats (total volume in the cortex: *P* < 0.001; total volume in the hippocampus: *P* < 0.001). We also assessed the level of soluble and insoluble Aβ1-40 and Aβ1-42 in the cortex and hippocampus. As shown in **Figure 4C**, the level of total soluble and total insoluble Aβ1-40 and Aβ1-42 in the rat AD brain were significantly decreased after PBM treatment (soluble Aβ1-40 in the cortex: *P* = 0.0003; soluble Aβ1-40 in the cortex: *P* = 0.0018; Insoluble Aβ1-40 in the cortex: *P* = 0.001; Insoluble Aβ1-40 in the cortex: *P* = 0.021; soluble Aβ1-42 in the cortex: *P* = 0.015; soluble Aβ1-42 in the cortex: *P* = 0.001; Insoluble Aβ1-42 in the cortex: *P* = 0.0107; Insoluble Aβ1-42 in the cortex: *P* = 0.0016). We next analyzed another key hallmark of AD, abnormal tau hyperphosphorylation, using immunostaining. As shown in **Figure 4D**, results of confocal microscopy revealed a significantly increased PHF intensity in both the cortex (*P* < 0.001) and hippocampus (*P* < 0.001) of AD animals as compared to WT animals, and this increased PHF intensity in the AD rats was significantly ameliorated by PBM treatment (Cortex: *P* = 0.0047; Hippocampus: *P* = 0.0038). No significant differences between WT animals with or without PBM treatment were detected.

### PBM treatment recruits microglia surrounding amyloid plaques by astrocytic IL-3 and microglial IL-3Rα

**Figure 5:**
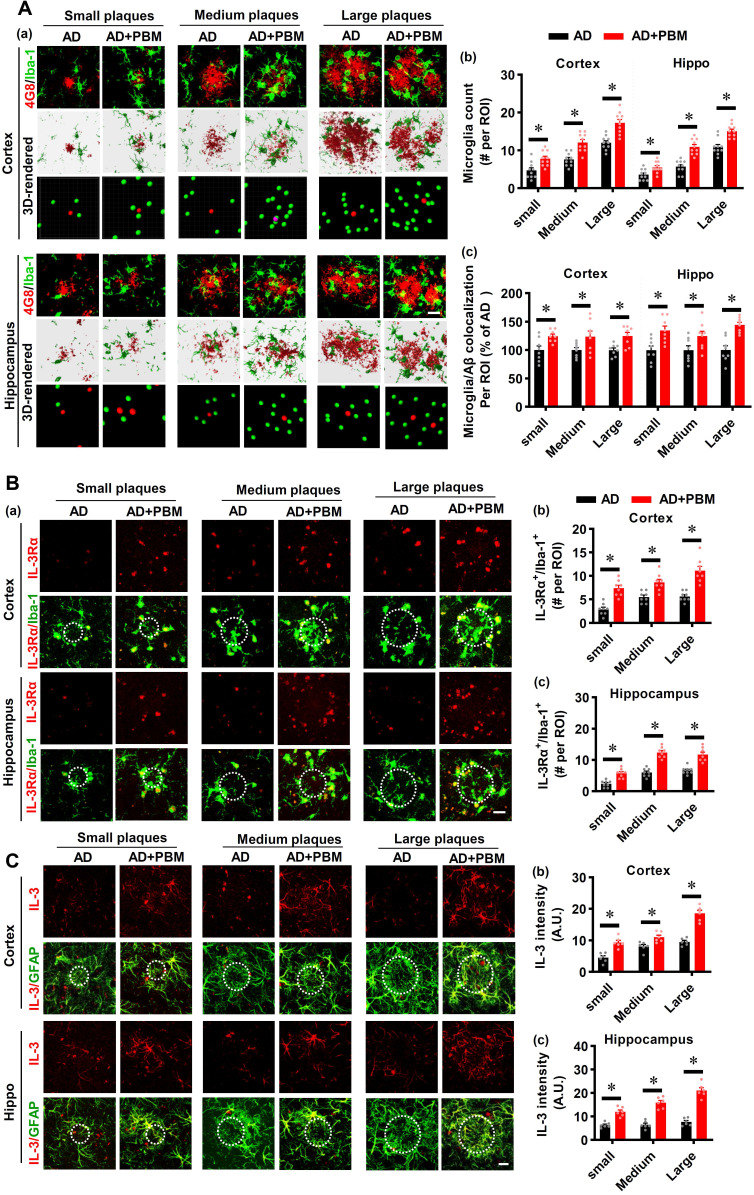
** PBM treatment recruits microglia surrounding amyloid plaques by regulating astrocytic IL-3 and microglial IL-3Rα. (A)** Representative immunofluorescence staining and 3D reconstruction of Iba-1 (green) and Aβ (4G8, red) in both cortex and hippocampus (a). Microglial count around amyloid plaques (b) and the colocalization between microglia and Aβ deposition (c) were analyzed. **(B)** Representative immunofluorescence images showing co-localization of IL-3Rα (red) and Iba-1 (green) in proximity to amyloid plaques in both the cortex and hippocampus (a). The number of IL-3Rα-positive microglia in the cortex (b) and (c) was analyzed. **(C)** Representative images showing co-localization of IL-3 (red) and GFAP (green) in both the cortex and hippocampus. Scale bar = 20 µm. Data are presented as mean ± SEM (n = 6). *P < 0.05.

Microglia, as the primary innate immune cells in the brain, play an important role in Aβ clearance 39. Therefore, Iba-1 staining was performed to investigate the microglial response in AD rats after PBM treatment. As shown in **Figure 5A**, the numbers of microglia surrounding the different sizes of amyloid plaques were analyzed. The results depicted in **Figure 5A** show that AD rats with PBM treatment displayed significantly increased microglia surrounding amyloid plaques compared to AD animals. Astrocytic IL-3 and microglial IL-3Rα play a critical role in the recruitment of microglia surrounding amyloid plaques 39. Therefore, we next assessed the levels of IL-3 and IL-3Rα in both the cortex and hippocampus. As shown in **Figure 5B**, IL-3Rα was strongly expressed and co-localized with Iba-1 surrounding amyloid plaques in both the cortex (Small: *P* < 0.001; Medium: *P* = 0.001; Large: *P* < 0.001) and hippocampus (Small: *P* < 0.001; Medium: *P* < 0.001; Large: *P* < 0.001) of AD animals with PBM treatment. Additionally, as shown in **Figure 5C**, representative images of the double immunofluorescence staining for GFAP and IL-3 showed significantly increased expression of IL-3 in the astrocytes around amyloid plaques of PBM-treated rats (Cortex-small: *P* = 0.0003; Cortex-medium: *P* = 0.008; Cortex-large: *P* < 0.001; Hippocampus-small: P = 0.0001; Hippocampus-medium: P < 0.001; Hippocampus-large: P < 0.001). No significant differences between WT animals with or without PBM treatment were detected. Similar results with PBM were obtained *in vitro* using purified astrocytes and microglia treated with Aβ1-42 (**Figure S4**).

### PBM treatment regulates the phenotype of glial cells and suppresses neuroinflammation

**Figure 6:**
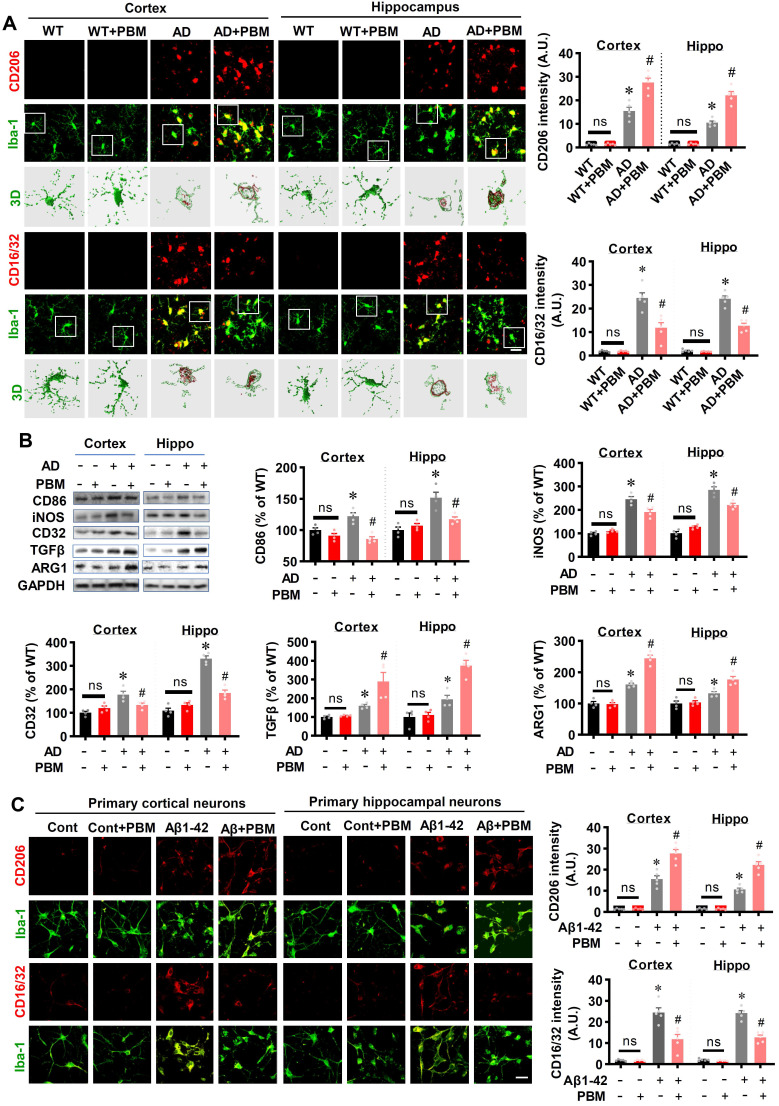
** PBM treatment promotes microglial polarization from M1 to M2 phenotype. (A)** Representative confocal microscopy images and 3D reconstruction images of Iba-1 with M1 marker CD16/32 or the M2 marker CD 206 in both the cortex and hippocampus. The relative fluorescent intensities of CD16/32 and CD206 were analyzed using Image J. Data are presented as mean ± SEM (n = 5). Rectangles indicate cells enlarged and 3D-rendered in the bottom row. Scale bar = 20 µm. **(B)** Western blotting analysis of M1 phenotype markers (i.e., CD32, CD86, and iNOS) and M2 phenotype markers (i.e., TGFβ and ARG). Data are presented as mean ± SEM (n = 4). **(C)** Immunofluorescence staining of Iba-1 with M1 marker CD16/32 or the M2 marker CD 206 *in vitro* cell culture. Scale bar = 20 µm. Data are presented as mean ± SEM (n = 6). *P < 0.05 versus WT group, ^#^ *P* < 0.05 versus AD or Aβ1-42 group.

**Figure 7:**
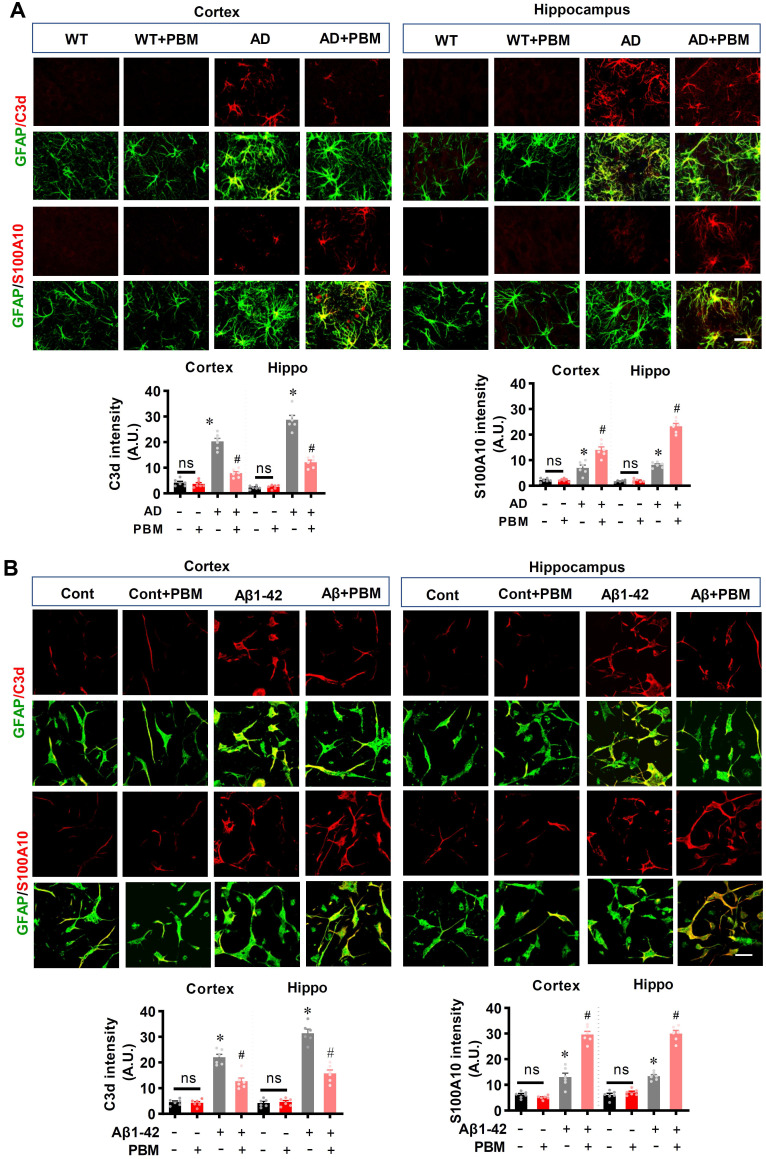
** PBM treatment promotes astrocytic polarization from A1 to A2 phenotype. (A)** Representative confocal microscopy images of GFAP (green) with A1 marker C3d or the A2 marker S100A10 in both the cortex and hippocampus. The relative fluorescent intensities of C3d and S100A10 were analyzed using Image J. **(B)** Representative confocal microscopy images of GFAP with C3d or S100A10 *in vitro* cell culture. Scale bar = 20 µm. Data are presented as mean ± SEM (n = 6). *P < 0.05 versus WT group, ^#^ *P* < 0.05 versus AD or Aβ1-42 group. ns indicates no significant difference (*P* > 0.05).

Neuroinflammation and glial cell polarization has been shown to play a pivotal role in the pathogenesis of AD 40-42. Therefore, we next investigated whether PBM treatment could affect neuroinflammation and the phenotype of glial cells. As shown in **Figure 6A**, double staining of CD206, an M2 phenotype marker, with Iba-1 showed that CD206^+^/Iba-1^+^ cells were significantly increased in the cortex and hippocampus of AD animals with PBM treatment (Cortex: *P* = 0.0012; Hippocampus: *P* = 0.00013). In contrast, representative images of the double staining CD16/32, an M1 phenotype marker, with Iba-1 showed that CD16/32^+^/Iba-1^+^ cells in the cortex and hippocampus of PBM-treated AD rats were significantly decreased (Cortex: *P* = 0.00385; Hippocampus: *P* = 0.00012). To further confirm these results, Western blotting using protein samples from the cortex and hippocampus was performed. As shown in **Figure 6B**, AD animals displayed significantly increased levels of M1 markers (i.e., CD86, iNOS, and CD32), and PBM treatment significantly alleviated this increase (Cortex-CD86: *P* = 0.0017; Hippocampus-CD86: *P* = 0.0121; Cortex-iNOS: *P* = 0.0133; Hippocampus-iNOS: *P* = 0.0063; Cortex-CD32: *P* = 0.035; Hippocampus-CD32: *P* = 0.0001). Consistent with these results, the M2 markers (i.e., TGFβ, ARG) in the PBM-treated group were significantly elevated compared with AD groups (Cortex-TGF-β: *P* = 0.040; Hippocampus- TGF-β: *P* = 0.0025; Cortex-ARG: *P* = 0.0003; Hippocampus-ARG: *P* = 0.0095). Primary microglial cell culture further confirmed the effect of PBM on the polarization of microglia (**Figure 6C**). As shown in **Figure 6C**, the levels of CD206 were significantly elevated by PBM treatment (Cortex: *P* = 0.00115; Hippocampus: *P* = 0.0001), and the levels of CD16/32 were significantly decreased in PBM-treated groups (Cortex: *P* = 0.0038; Hippocampus: *P* = 0.00012). Next, the effects of PBM treatment on astrocyte phenotypes were also analyzed. **Figure 7A** showed that C3d (the neurotoxic A1 marker) expression was significantly increased in AD rats compared to WT animals (Cortex: *P* < 0.001; Hippocampus: *P* < 0.001) and PBM treated animals (Cortex: *P* < 0.001; Hippocampus: *P* < 0.001). In contrast, expression of the A2 marker S100A10 was significantly elevated by PBM treatment in AD animals (Cortex: *P* = 0.0012; Hippocampus: *P* < 0.001), suggesting that the PBM enhance the neuroprotective A2 phenotype. Primary microglial cell cultures further confirmed these results (**Figure 7B**). No significant differences between the WT group with or without PBM treatment were detected.

Next, we investigated the effects of PBM treatment on inflammatory cytokines. As shown in **Figure S5**, the levels of pro-inflammatory cytokines (i.e., NFκB, TNF-α, and IL-1β) were significantly increased in the cortex and hippocampus of AD rats (Cortex-NFκB: *P* = 0.00173; Hippocampus-NFκB: *P* < 0.001; Cortex-TNF-α: *P* = 0.0005; Hippocampus-TNF-α: *P* = 0.0179; Cortex-IL-1β: *P* < 0.001; Hippocampus-IL-1β: *P* < 0.001). PBM treatment, however, significantly ameliorates the levels of pro-inflammatory cytokines (Cortex-NFκB: *P* = 0.00139; Hippocampus-NFκB: *P* = 0.0057; Cortex-TNF-α: *P* = 0.0154; Hippocampus-TNF-α: *P* = 0.035; Cortex-IL-1β: *P* < 0.001; Hippocampus-IL-1β: *P* < 0.001). Furthermore, the anti-inflammatory cytokines (IL-4, IL-10, and IL-13) were significantly elevated in the cortex and hippocampus of AD rats rats (Cortex-IL-4: *P* = 0.0054; Hippocampus-IL-4: *P* = 0.0001; Cortex-IL-10: *P* = 0.0004; Hippocampus-IL-10: *P* < 0.001; Cortex-IL-13: *P* = 0.0005; Hippocampus-IL-13: *P* < 0.001), and PBM treatment further enhanced the levels of these anti-inflammatory cytokines (Cortex-IL-4: *P* = 0.0007; Hippocampus-IL-4: *P* = 0.0002; Cortex-IL-10: *P* = 0.0004; Hippocampus-IL-10: *P* = 0.025; Cortex-IL-13: *P* = 0.0054; Hippocampus-IL-13: *P* = 0.0068). No significant differences between the WT group with or without PBM treatment were detected.

### PBM treatment suppresses mitochondrial fragmentation, preserves mitochondrial dynamics, and alleviates oxidative stress

**Figure 8:**
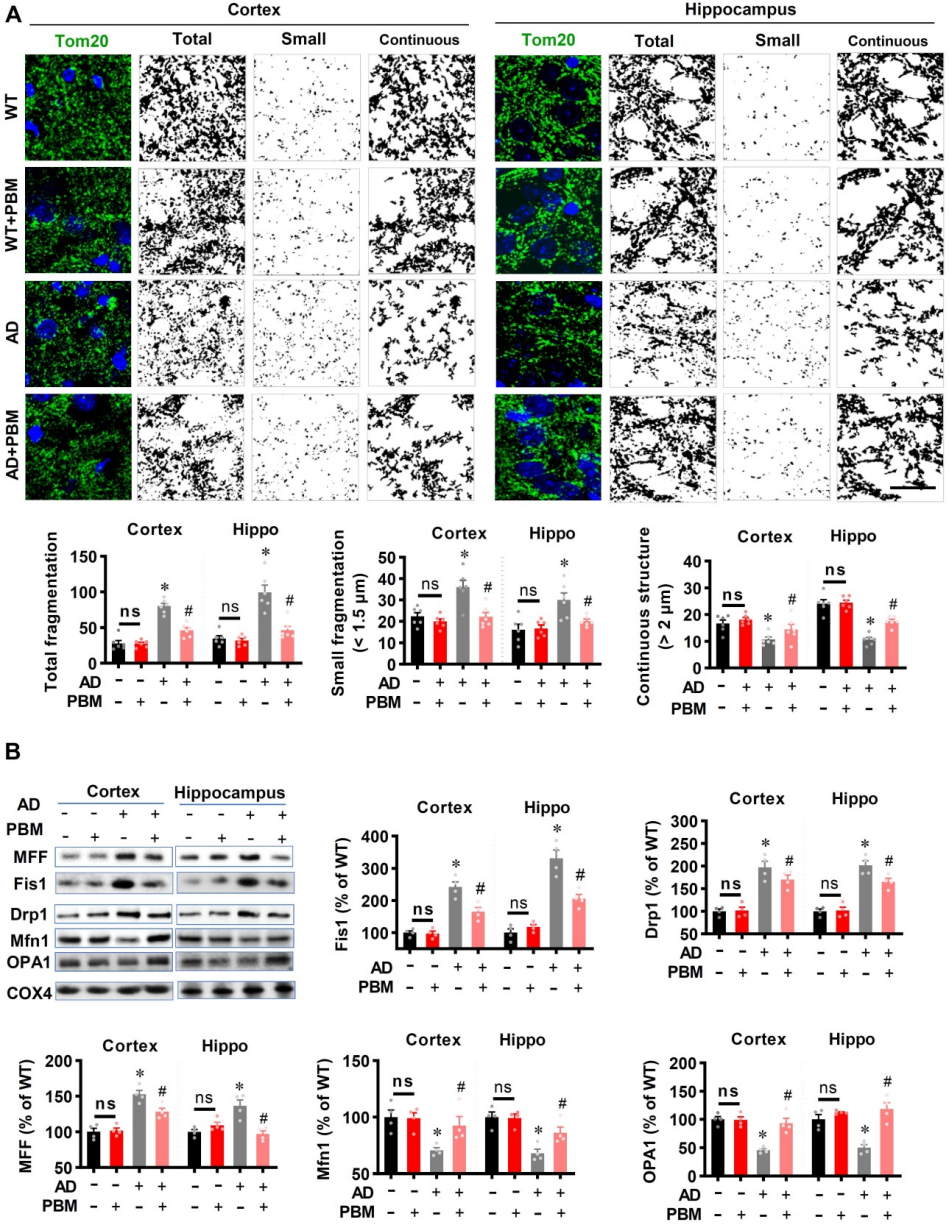
** PBM treatment preserves mitochondrial dynamics and regulates mitochondrial fission and fusion-associated proteins in the cortex and hippocampus. (A)** Representative confocal microscopy images of Tom20 (green, a mitochondrial outer membrane marker) and DAPI in the cortex and hippocampus. The images were processed using Image J, and the mitochondrial segments were separated as total particles, small particles (size < 1.5 µm), and continuous structures (size > 2 µm). The number of total particles, small particles, and continuous structures was normalized using the total mitochondrial area. **(B)** Western blotting and quantitative analyses of mitochondrial fission proteins (i.e., MFF, FIS1, Drp1) and fusion proteins (i.e., MFN2 and OPA1). Data are presented as mean ± SEM (n = 4). *P < 0.05 versus WT group, ^#^ *P* < 0.05 versus AD group. ns indicates no significant difference (*P* > 0.05).

Excessive mitochondrial fragmentation is well documented in Alzheimer's disease 43, and closely related to neuroinflammation and oxidative stress 44. Therefore, we next investigated the effect of PBM treatment on mitochondrial fragmentation. As shown in** Figure 8A**, AD animals displayed significantly increased total fragmentation (Cortex: *P* < 0.001; Hippocampus: *P* = 0.00016) and small fragmentation (Cortex: *P* = 0.0038; Hippocampus: *P* = 0.007) in both the cortex and hippocampus as compared with WT animals. In contrast, the continuous structure was significantly decreased in AD animals compared with WT animals (Cortex: *P* = 0.0042; Hippocampus: *P* < 0.001). Intriguingly, PBM treatment significantly suppresses mitochondrial fragmentation. To further confirm these results, mitochondrial dynamics-related proteins were analyzed using Western blotting. As shown in **Figure 8B**, the expression of mitochondrial fission proteins (i.e., MFF, Fis1, and Drp1) was significantly increased in both the cortex and hippocampus of AD rats compared to WT animals (Cortex-MFF: *P* = 0.0004; Hippocampus-MFF: *P* = 0.0007; Cortex-Fis1: *P* = 0.0002; Hippocampus-Fis1: *P* = 0.0002; Cortex-Drp1: *P* = 0.0005; Hippocampus-Drp1: *P* = 0.001). In contrast, PBM treatment alleviated these increases (Cortex-MFF: *P* = 0.0188; Hippocampus-MFF: *P* = 0.0069; Cortex-Fis1: *P* = 0.0095; Hippocampus-Fis1: *P* = 0.0005; Cortex-Drp1: *P* = 0.16; Hippocampus-Drp1: *P* = 0.031). In addition, the expression of mitochondrial fusion proteins (i.e., Mfn1 and OPA1) was significantly decreased in the cortex and hippocampus of AD animals (Cortex-Mfn1: *P* = 0.005; Hippocampus-Mfn1: *P* = 0.0014; Cortex-OPA1: *P* < 0.001; Hippocampus-OPA1: *P* = 0.00264), and significantly preserved by PBM treatment (Cortex-Mfn1: *P* = 0.0038; Hippocampus-Mfn1: *P* = 0.0029; Cortex-OPA1: *P* = 0.0026; Hippocampus-OPA1: *P* = 0.0015). To determine the effect of PBM on oxidative stress and antioxidant capacity, the products of oxidative stress were analyzed using assay kits and immunostaining. As shown in **Figure S6A**, total antioxidant capacity was significantly decreased in both the cortex and hippocampus of AD rats compared to WT animals (Cortex: *P* = 0.0056; Hippocampus: *P* = 0.028) and PBM-treated rats (Cortex: *P* = 0.0065; Hippocampus: *P* = 0.021). In addition, as shown in **Figure S6B-E,** the levels of MDA (lipid peroxidation), protein carbonyls, 8-OHdG (oxidized DNA damage), and 4HNE (lipid peroxidation) were significantly increased in AD animals compared with WT animals, which were significantly attenuated by PBM treatment. No significant differences between the WT group with or without PBM treatment were detected.

### PBM Treatment Preserves Neuronal Hemoglobin

**Figure 9:**
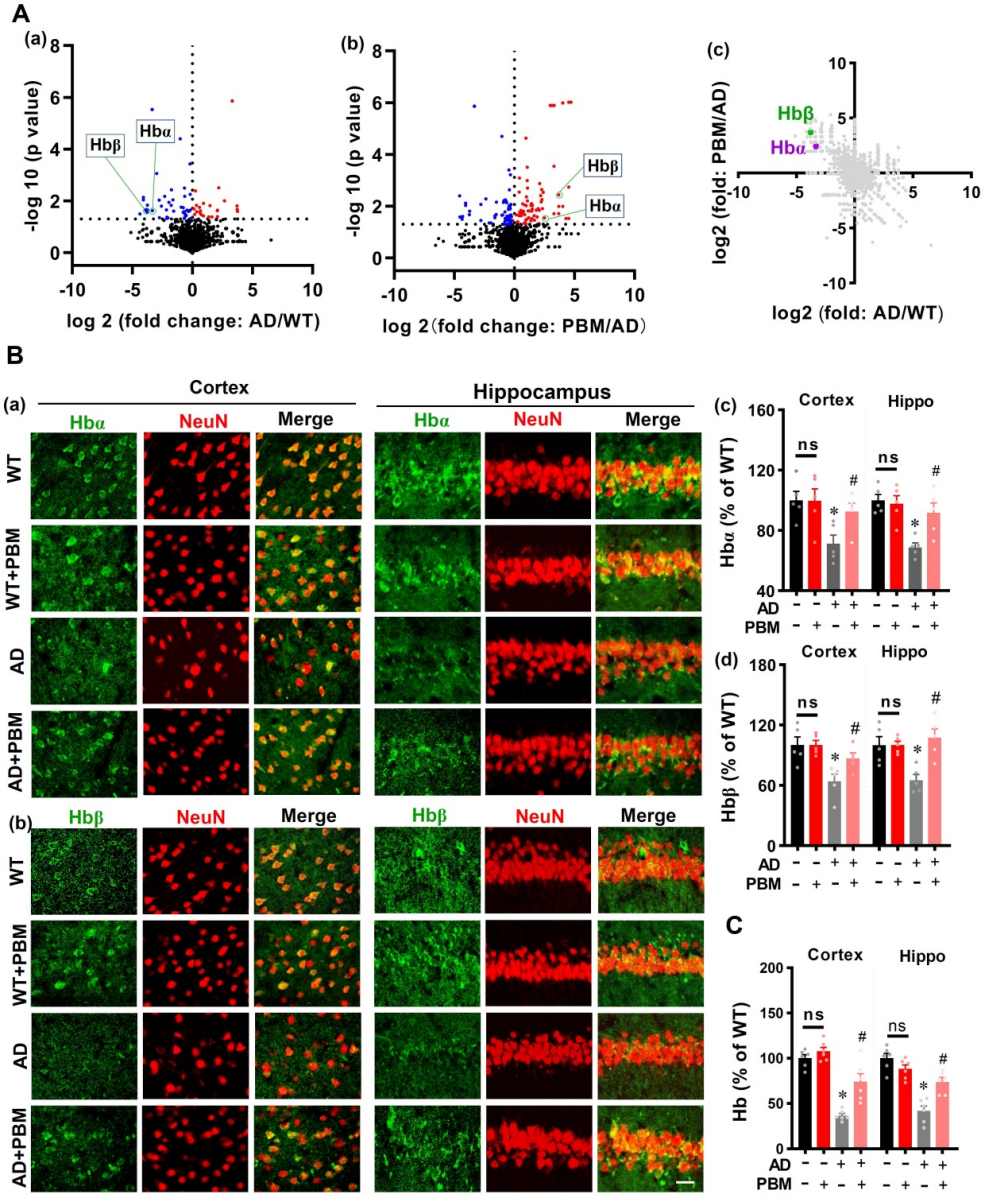
** PBM Treatment preserves neuronal hemoglobin. (A)** The volcano plot of mass spectrometry results. The blue/red dots indicate the decreased/increased level of proteins in the AD group when compared with the WT group (a). The volcano plot in (b) shows the results of PBM comparing with the AD group. The level of Hbα and Hbβ were significantly decreased in AD compared with WT animals, which were preserved in AD animals with PBM treatment. The dotted line indicates *P* < 0.05. (c) A two-dimension plot for the ratio of AD/WT and PBM/AD. n=3. **(B)** Representative images showing co-localization of Hbα (green in (a)) or Hbβ (green in (b)) and GFAP (red) in both the cortex and hippocampus. The fluorescent intensities of Hbα (c) and Hbβ (d) were analyzed. Scale bar = 20 µm. **(C)** The levels of hemoglobin were measured by a hemoglobin colorimetric assay kit. Data are presented as mean ± SEM (n = 6). *P < 0.05.

Next, mass spectrometry was performed to detect the significant proteins in AD animals (AD vs WT) and AD animals with PBM treatment (PBM vs AD). As shown in **Figure 9A (a and b)**, the volcano plot showed a significantly decreased level of hemoglobin α (Hbα) and β (Hbβ) in the brain tissue of AD animals compared with WT animals (Hbα: *P* = 0.024; Hbβ: *P* = 0.023), which were preserved in AD animals with PBM treatment (Hbα: *P* = 0.049; Hbβ: *P* = 0.0037). A two-dimension plot for the ratio of AD/WT and PBM/AD further confirmed these results (**Figure 9A(c)**). Therefore, we further examined the effect of PBM treatment on neuronal hemoglobin using immunofluorescence staining. As shown in **Figure 9B**, representative confocal microscopy images showed a robust decrease of Hbα and Hbβ in both the cortex and hippocampus of AD animals compared to WT (Hbα in the cortex: *P* = 0.0079; Hbα in the hippocampus: *P* = 0.0002; Hbβ in the cortex: *P* = 0.0105; Hbβ in the hippocampus: *P* = 0.0.0086). Notably, PBM treatment was able to significantly alleviate these changes in AD rats (Hbα in the cortex: *P* = 0.028; Hbα in the hippocampus: *P* = 0.013; Hbβ in the cortex: *P* = 0.033; Hbβ in the hippocampus: *P* = 0.0036). To further confirm these results, the level of hemoglobin was also examined using a hemoglobin colorimetric detection kit. As shown in **Figure 9C**, the levels of hemoglobin in both the cortex and hippocampus of AD animals were significantly decreased compared to WT animals (Cortex: *P* < 0.001; Hippocampus: *P* < 0.001). In contrast, PBM treatment significantly alleviated this decrease (Cortex: *P* = 0.0023; Hippocampus: *P* = 0.0013). No significant differences between the WT group with or without PBM treatment were detected. Results from *in vitro* primary cell culture also confirmed these results. As shown in **Figure S7**, the expression of hemoglobin α was significantly decreased in the toxic Aβ1-42 group (Cells from the cortex: *P* < 0.001; Cells from the hippocampus: *P* < 0.001), and the levels of hemoglobin in both the cortex and hippocampus were preserved in neurons with PBM treatment (Cells from the cortex: *P* < 0.001; Cells from the hippocampus: *P* < 0.001). Western blotting analysis of hemoglobin further confirmed these results. No significant differences between the WT group with or without Aβ42-1 (no toxic) were detected.

### Neuronal hemoglobin knockdown suppresses the neuroprotective effect of PBM treatment

**Figure 10:**
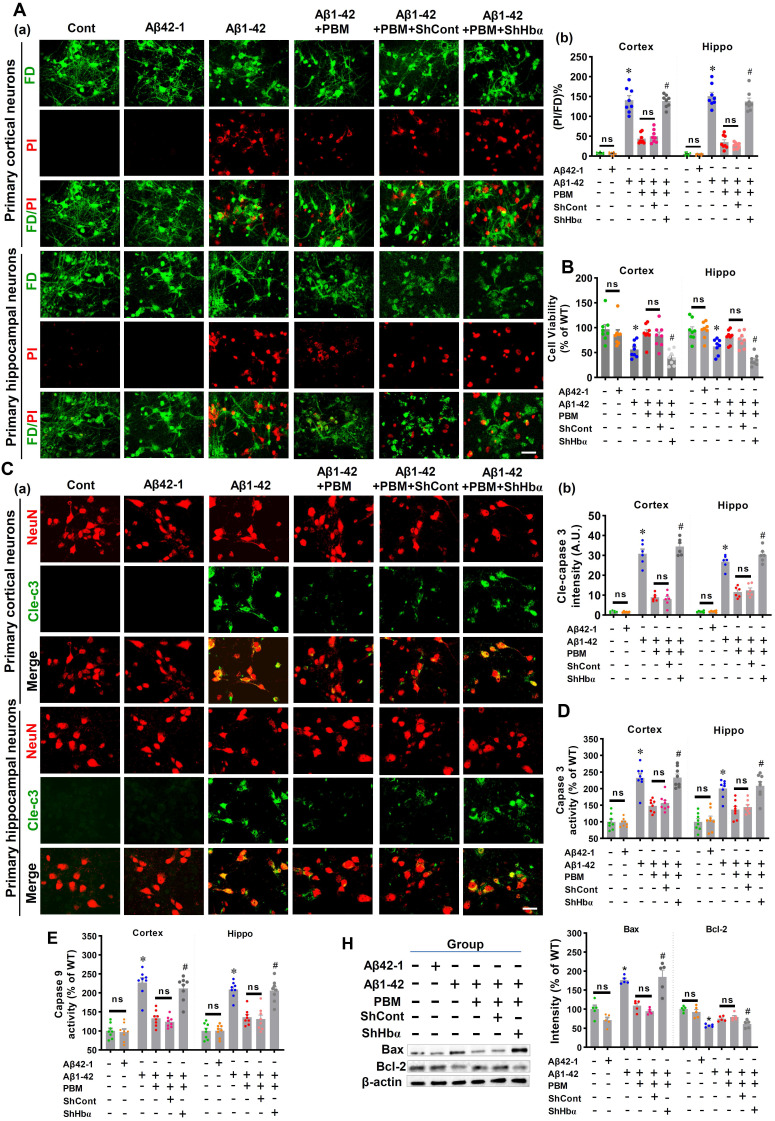
** Hbα knockdown abolishes PBM treatment's protection on primary culturing neurons. (A)** Cell viability was measured using dual-fluorescent FD/PI assay. (a) Representative confocal microscopy images of FD/PI staining of primary cortical neurons and hippocampal neurons. Cellular viability was expressed as the ratio of FD/PI (n=8). Scale bar = 20 µm. **(B)** MTT assay was performed to test cellular metabolic activity as an indicator of cell viability. Data was quantified as percentage changes versus the cont. group. **(C)** Levels of cleaved-caspase 3 were measured using immunofluorescence staining. Scale bar = 20 µm. n=6. **(D)** The caspase-3 and **(E)** caspase 9 activity was measured using the corresponding activity assay kit and expressed as a percentage change versus the control group (n=8). **(H)** Western blotting and quantitative analyses of Bax and Bcl-1. Data were presented as a percentage change versus the control group (n=5). *P < 0.05 versus control group, ^#^ *P* < 0.05 versus PBM group. ns indicates no significant difference (*P* > 0.05).

Using primary neuronal cell culture, we next investigated the role of neuronal hemoglobin in PBM's beneficial effects. As shown in **Figure S7A and B,** the levels of Hbα were significantly knocked down in the primary neuronal cells from both the cortex and hippocampus compared to the scrambled shRNA group, demonstrating that the Hbα shRNA used in our project was capable of efficient knockdown of Hbα. To validate the role of Hbα in mediating the protective effect of PBM on neurons, cortical neurons and hippocampal neurons were double labeled with FD (green, viable cells) and PI (red, dead cells). Our data showed that Aβ1-42 induced significantly increased PI-positive cells compared to control cells (Cortex: *P* < 0.001; Hippocampus: *P* < 0.001) and PBM-treated cells (Cortex: *P* < 0.001; Hippocampus: *P* < 0.001) (**Figure 10A**). However, Hbα knockdown abolished the neuroprotective effect of PBM treatment (Cortex: *P* < 0.001; Hippocampus: *P* < 0.001) (**Figure 10A**). MTT assay results shown in **Figure 10B** further confirmed that the neuronal cell viability was significantly decreased after the addition of Aβ1-42 compared with the control group (Cortex: *P* = 0.0023; Hippocampus: *P* = 0.004). In contrast, PBM treatment protected neurons against Aβ1-42-induced neurotoxicity (Cortex: *P* = 0.0018; Hippocampus: *P* < 0.001). Interestingly, it was noted that the protective effect of PBM on neurons was lost in the Hbα knockdown group (Cortex: *P* < 0.001; Hippocampus: *P* < 0.001). In addition, immunostaining for cleaved caspase 3 was performed. Neurons exposed to Aβ1-42 displayed a significantly increased level of cleaved caspase 3 (Cortex: *P* < 0.001; Hippocampus: *P* < 0.001), which was alleviated by PBM treatment (Cortex: *P* < 0.001; Hippocampus: *P* < 0.001) (**Figure 10C**). Intriguingly, the beneficial effect of PBM on neurons was not found in the Hbα knockout group (**Figure 10C**). To further confirm these results, the activity of caspase 3 and caspase 9 were measured using the corresponding fluorometric assay kit. As shown in **Figure 10D and E**, the activities of caspase 3 and caspase 9 were significantly increased after Aβ1-42 compared with the control group (Cortex-caspase 3: *P* < 0.001; Hippocampus-caspase 3: *P* < 0.001; Cortex-caspase 9: *P* <0.001; Hippocampus-caspase 9: *P* < 0.001) and PBM group (Cortex-caspase 3: *P* < 0.001; Hippocampus-caspase 3: *P* < 0.003; Cortex-caspase 9: *P* <0.001; Hippocampus-caspase 9: P < 0.001), and Hbα knockdown abolished the effect of PBM on caspase 3 and caspase 9. The expressions of Bax and Bcl-2 were consistent with these results (**Figure 10H**). Furthermore, the expressions of PSD95 and spinophilin were significantly reduced in the Aβ1-42-treated group compared with the control group (Cortex-PSD-95: P < 0.001; Hippocampus-PSD-95: *P* < 0.001; Cortex-spinophilin: *P* < 0.001; Hippocampus spinophilin: *P* < 0.001) and the PBM-treated group (Cortex-PSD-95: P = 0.0011; Hippocampus-PSD-95: P = 0.0039; Cortex-spinophilin: *P* = 0.0068; Hippocampus spinophilin: *P* = 0.0092). Notably, the effect of PBM treatment was suppressed by Hbα knockdown (**Figure S8**).

### Neuronal hemoglobin knockdown suppresses the effect of PBM on mitochondria and intraneuronal oxygen homeostasis

**Figure 11:**
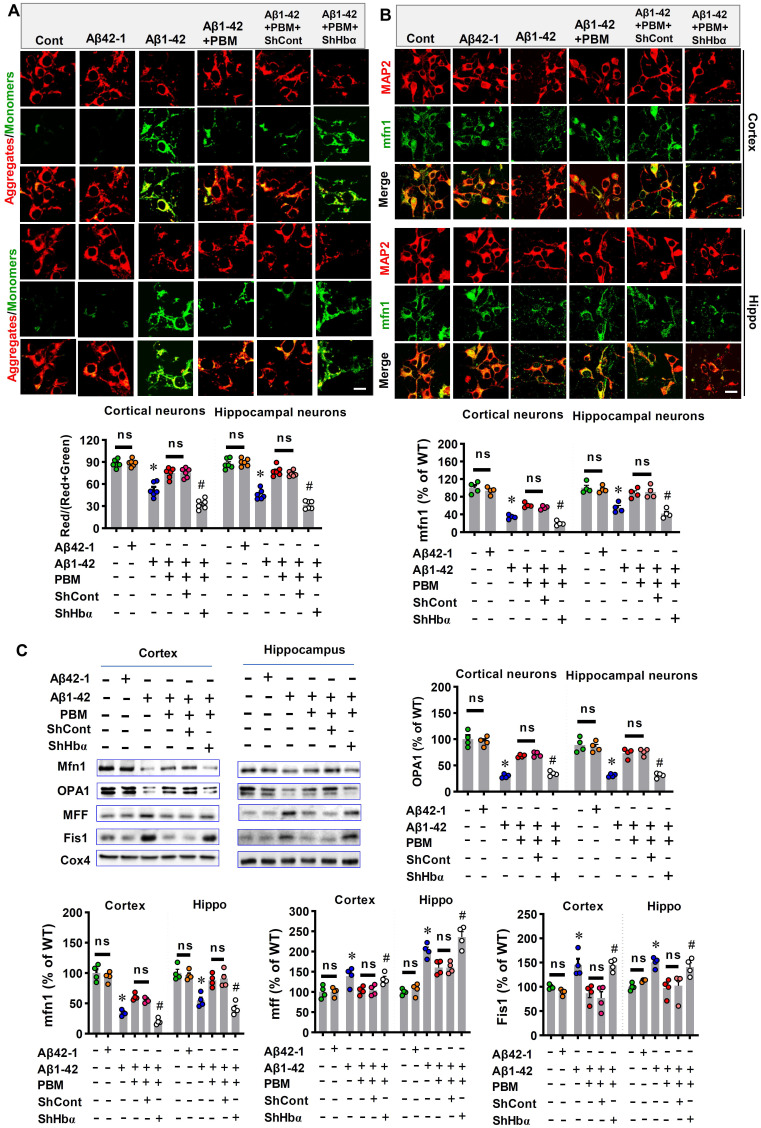
** Hbα knockdown abolishes PBM treatment's protection on mitochondria in the primary culturing neurons. (A)** JC-1 Dye was used for detecting mitochondrial membrane potential in the primary culturing neurons. The red JC-1 aggregates indicate high mitochondrial membrane potential, and the green JC-1 monomers indicate low mitochondrial membrane potential. **(B)** The primary culturing neurons were stained with MAP2 and mfn2 antibodies. The intensities of mitochondrial fusion-related mfn2 were expressed percentage changes versus the control group. Scale bar = 10 µm. **(C)** Western blotting and quantitative analyses of mitochondrial fission proteins (i.e., MFF and FIS1) and fusion proteins (i.e., MFN2 and OPA1). All data were expressed as a percentage change versus the control group and presented as mean ± SEM (n = 4-6). *P < 0.05 versus cont. group, ^#^ *P* < 0.05 versus PBM group. ns indicates no significant difference (*P* > 0.05).

We finally analyzed the role of neuronal hemoglobin in the effect of PBM on mitochondria and intraneuronal oxygen homeostasis. First, the JC-1 assay kit was used to measure the mitochondrial membrane potential. The green fluorescence and red fluorescence represented low and high mitochondrial membrane potential (MMP), respectively. As shown in **Figure 11A**, Aβ1-42 exposure displayed significantly increased green fluorescence intensity and significantly decreased red/(red+green) ratio compared with the control group (Cortex: *P* < 0.001; Hippocampus: *P* < 0.001). In contrast, PBM treatment significantly alleviated the Aβ1-42-induced increase of green fluorescence (Cortex: *P* = 0.00094; Hippocampus: *P* < 0.001), suggesting PBM treatment preserved the MMP after the Aβ1-42 exposure. Notably, Hbα knockdown suppressed the effect of PBM treatment on the MMP of neurons from both the cortex (*P* < 0.001) and hippocampus (*P* < 0.001). Next, we analyzed the role of neuronal hemoglobin in the effect of PBM on mitochondrial fusion and fission. As shown in **Figure 11B and C**, the expression of mitochondrial fusion proteins (i.e., MFN1 and OPA1) were significantly reduced after Aβ1-42 exposure while the expression of mitochondrial fission proteins (i.e., MFF and Fis1) were significantly increased compared with the control group (Cortex-MFN1: *P* < 0.001; Hippocampus-MFN1: *P* = 0.00015; Cortex-OPA1: *P* < 0.001; Hippocampus-OPA1: *P* = 0.00151; Cortex-MFF: *P* = 0.031; Hippocampus-MFF: *P* < 0.001; Cortex-Fis1: *P* = 0.0117; Hippocampus-Fis1: *P* = 0.0002) and PBM group (Cortex-MFN1: *P* < 0.001; Hippocampus-MFN1: *P* = 0.0001; Cortex-OPA1: *P* = 0.0003; Hippocampus-OPA1: *P* = 0.00475; Cortex-MFF: *P* = 0.029; Hippocampus-MFF: *P* = 0.0138; Cortex-Fis1: *P* = 0.0097; Hippocampus-Fis1: *P* = 0.00264). It is notable that Hbα knockdown significantly suppressed these changes with PBM treatment. Finally, using a hypoxyprobe™-1 kit, we measured the role of hemoglobin of intraneuronal oxygen homeostasis. As shown in **Figure S9,** Aβ1-42 induced a significantly increased fluorescence intensity of hypoxyprobe-1 (Cortex: *P* < 0.001; Hippocampus: *P* < 0.001), suggesting Aβ1-42 induced intraneuronal hypoxia, an effect significantly ameliorated by PBM treatment (Cortex: *P* < 0.001; Hippocampus: *P* < 0.001). In contrast, the effect of PBM treatment was suppressed in the Hbα knockdown group (Cortex: *P* < 0.001; Hippocampus: *P* < 0.001), suggesting Hbα plays an important role in mediating the beneficial effect of PBM treatment.

## Discussion

Our studies demonstrate that long-term PBM treatment with an 808 nm low-level laser can attenuate cognitive dysfunction in AD-like rats and exerts a neuroprotective effect against neuronal damage and loss. In addition, we found that PBM treatment protects against characteristic pathophysiological features of AD, including attenuation of amyloid plaque deposition and abnormal Tau hyperphosphorylation. Our molecular studies further revealed that PBM treatment: 1) recruits microglia surrounding amyloid plaques and improves microglial Aβ clearance by enhancing the expression of astrocytic IL-3 and microglial IL-3Rα; 2) regulates microglial and astrocytic phenotype from a neurotoxic (Microglial M1 and astrocytic A1 phenotype) to a neurotrophic phenotype (Microglial M2 and astrocytic A2 phenotype) and inhibits neuroinflammation; 3) preserves mitochondrial dynamics and alleviates oxidative stress; and 4) preserves neuronal hemoglobin. Notably, neuronal hemoglobin knockdown abolished the neuroprotective effect of PBM treatment. Taken together, our studies suggest that a multitude of factors contribute to the beneficial effect of PBM on AD, and that the mitochondria-hemoglobin pathway and regulation of glial cells play a central role in regulating these changes. Thus, our study supports the possible use of PBM treatment to prevent neurodegeneration in the early stages of AD and provides a novel mechanism of PBM-mediated neuroprotection.

Currently, the operation mode of PBM therapy can either be pulsed or continuous wave 45. Previous studies have found the beneficial effects of pulsed wave and continuous wave PBM therapy on the brain 21, 46-48. Currently, pulsed wave modes at 10-Hz 48, 40-Hz 16, and 100-Hz 45, 49 are the most widely used frequencies. PBM therapy at pulsed mode was able to protect against brain injury 22, attenuates Alzheimer's-associated pathology, and improves learning and memory 16. The underlying mechanism of pulsed wave mode includes the regulation of microglia, gamma rhythms, mitochondrial membrane ion channels. Similarly, the neuroprotective effect of PBM therapy has been reported in various brain diseases 6, 45. As mentioned previously, the most well-accepted mechanism of continuous wave PBM therapy centers around CCO 6, 45, 50. According to previous studies, light/laser in pulsed wave mode may have better penetration depth and be more effective than continuous wave mode 45, 48. However, the effects of PBM therapy rely on multiple parameters and further work is still needed to define the effects of PBM therapy with different operation mode for different brain disease 51.

Recently, several studies demonstrated the beneficial role of PBM treatment with pulsed wave light in AD 16, 52, 53. However, few studies have focused on investigating the effects of long-term PBM therapy with continuous-wave laser at the early disease progression. In the present study, we found 18-month-old TgF344-AD rats displayed significant cognitive dysfunction, including spatial learning and memory, short-term working memory, and recognition memory, consistent with previous studies 24, 54. Interestingly, using a long-term PBM therapy with 808 nm continuous-wave near-infrared laser, we demonstrated that PBM therapy starting from two months of age alleviated cognitive impairment in AD-like rats, indicating PBM treatment could slow the cognitive impairment during AD progression. In addition, our studies also found long-term PBM treatment at the early stage of AD was able to reduce Aβ deposition, abnormal tau hyperphosphorylation, neuronal damage, neuronal apoptosis, neuronal degeneration, and protect spine synapses and dendritic spine. These findings further confirmed the neuroprotective effect of the PBM treatment at the early stages of AD.

In the brain, microglia are considered as the first and main form of immune defense 55. However, in AD, the protective function of microglia is compromised 56, 57, as evidenced by their limited ability for Aβ clearance and increased microglial-mediated inflammation 3, 40, 58, 59. Microglial phagocytosis plays a crucial role in Aβ clearance 60, 61. However, microglia without exogenous stimulation or facilitation in AD displayed impaired Aβ clearance 58, 62, 63. In previous studies, 40-Hz light flicker therapy and PBM treatment with 1070-nm light pulsed at 10 Hz was able to recruit microglia and reduce amyloid levels 16, 52, 64. Here, our results indicated PBM treatment with continuous-wave low-level laser is also effective in the recruitment of microglia and improving Aβ clearance, suggesting PBM therapy with continuous-wave low-level laser has similar efficiency in regulating microglial recruitment and microglial phagocytosis.

As mentioned above, previous studies have demonstrated the ability of PBM therapy to regulate microglia recruitment to Aβ deposits. However, the underlying mechanism is unclear 16, 52, 64. In the current study, in both *in vivo* and *in vitro* studies, we demonstrated that PBM treatment can enhance the expression of astrocytic interleukin-3 and microglial IL-3Rα, the specific receptor for IL-3. Astrocytic IL-3 targeting of microglial IL-3Rα has been shown to enhance the capacity of microglia to cluster and clear Aβ 39. Thus, our finding provides a potential mechanistic explanation as to why PBM treatment was able to improve Aβ clearance in our study and in previous studies. In addition, we found PBM treatment was able to regulate microglial and astrocytic polarization. Microglial and astrocytic polarization was defined as the transition of microglia/astrocyte from the M1/A1 to M2/A2 phenotype 65. Microglial M1 and astrocytic A1 phenotype lose their ability to carry out their protective function, produce pro-inflammatory cytokines, and promote AD progression 65, 66. In contrast, microglial M2 and astrocytic A2 phenotypes are anti-inflammatory, releasing numerous trophic and protective factors to alleviate AD pathologies and improve phagocytotic function 65, 67, 68. Consistent with these findings, pro-inflammatory/anti-inflammatory cytokines were decreased/increased respectively, after PBM treatment.

Mitochondrial dysfunction is a common and prominent feature of neurodegenerative disease, including AD 69. Impaired mitochondrial fusion and excessive mitochondrial fission can induce mitochondrial fragmentation in AD, which contributes to the progression of AD 70, 71. In the current study, we found PBM treatment was able to alleviate excessive mitochondrial fission-induced mitochondrial fragmentation. According to a previous study, a healthy pool of mitochondria plays a central role in minimizing mitochondrial-associated oxidative damage 69. In our study, we found that PBM application significantly attenuated mitochondria-related oxidative damage, including damage to proteins, DNA, and lipids. Because mitochondrial function and oxidative stress are closely associated with neuroinflammation, the changes of mitochondria and oxidative stress after PBM treatment were consistent with the changes of glial cells and associated neuroinflammation.

Previous studies have found Hbα and Hbβ are expressed in neurons of both the rodent and human brain and have been implicated to play a critical role in maintaining normal mitochondrial function and intraneuronal oxygen homeostasis 72-74. Interestingly, neuronal hemoglobin is reduced in the hippocampus and frontal cortex of patients with AD 73, 75. In the current study, mass spectrometry also found Hbα and Hbβ expressions were significantly reduced in AD rats. However, the expression of Hbα and Hbβ were preserved by PBM treatment. Furthermore, PBM treatment was able to alleviate aβ1-42 induced hypoxia in neuronal cells, which is consistent with PBM preservation of neuronal hemoglobin. In addition to regulating neuronal oxygen homeostasis, neuronal hemoglobin plays an important role in the preservation of mitochondrial function in the brain 75, 76. In our *in vitro* study, we found that the PBM-induced preservation of mitochondrial membrane potential and mitochondrial dynamics was abolished by Hbα knockdown. Furthermore, we found that the neuroprotective role of PBM was also abolished by Hbα knockdown, as evidenced by decreased cell viability, increased neuronal apoptosis, and cellular damage. Taken together, these findings support a key role for neuronal hemoglobin in mediating the neuroprotective actions of PBM treatment.

**Figure 12:**
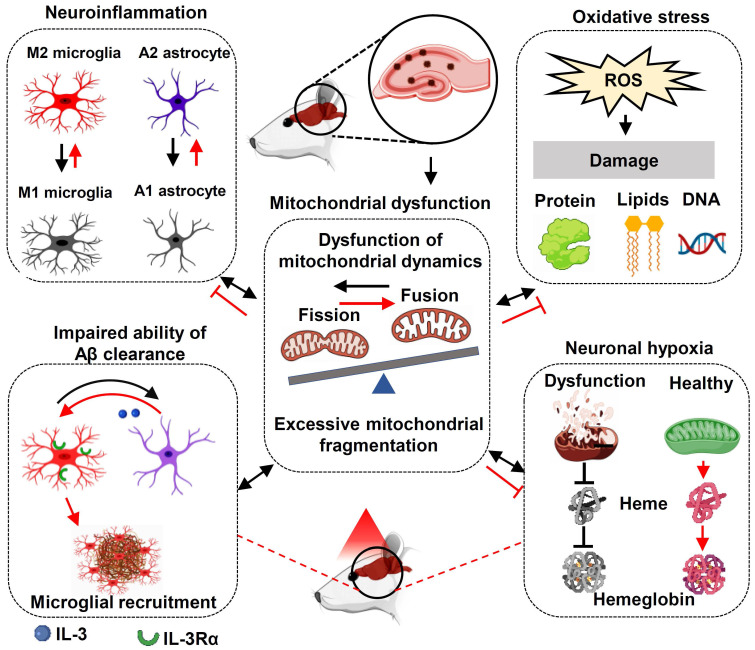
** Molecular mechanisms of PBM treatment in AD.** The improvement of multiple targets contributes to the beneficial effects of PBM treatment including the inhibition of oxidative damage and neuroinflammation; promoting M2 and A2 polarization; improving microglial recruitment; the preservation of mitochondrial function and mitochondrial integrity, and the expression of neuronal hemoglobin. Red lines indicate proposed beneficial effects/mechanisms of PBM in AD.

The failure of therapy targeting only one AD-related pathology suggests that multi-target therapy may be a better approach in the treatment of AD 77, 78. As shown in **Figure 12**, we found PBM could recruit microglia surrounding amyloid plaques, regulate glial cell phenotype, and preserve mitochondrial dynamics and the expression of neuronal hemoglobin, all of which are suggested to contribute to the neuroprotective role of PBM. Although multiple factors are involved in the beneficial effect of PBM on AD, these factors are closely related to each other. In our study, we found mitochondrial dynamics and mitochondrial function were preserved by PBM treatment, which was consistent with previous studies wherein mitochondria were considered as the main target of PBM 6, 20. In AD, mitochondrial alterations were able to induce oxidative damage, neuroinflammation, and glial cell polarization with M1/M2 and A1/A2 phenotype changes 79, 80. In our study, PBM treatment suppressed oxidative damage and neuroinflammation and promotes a shift from M1/A1 phenotype to M2/A2 phenotype, which may also depend on the preservation of mitochondrial function by PBM treatment. Moreover, because microglial phagocytosis requires a large amount of energy 63, and the M2 microglia possess an increased phagocytic activity 81, the preservation of mitochondrial function and the elevated M2 polarization by PBM treatment promotes the clearance of Aβ. In addition, heme, a major component of hemoglobin, is synthesized in the mitochondria 82. Thus, the synthesis of hemoglobin is also affected by mitochondrial function, and the decreased neuronal hemoglobin and increased neuronal hypoxia also modulates mitochondrial dynamics and function. Taken together, our findings demonstrate the beneficial effects of the long-term PBM therapy with 808 nm continuous-wave near-infrared laser on AD, and provide novel mechanistic insights into how PBM protects the brain against AD.

## Supplementary Material

Supplementary figures.

## Abbreviations

AD: Alzheimer's disease
PBM: Photobiomodulation
NFT: Neurofibrillary tangles
CCO: Cytochrome C oxidase
PTSD: Post-traumatic stress disorder
P1: Postnatal day 1
DIV: *In vitro* day 10
FBS: Fetal bovine serum
ELISA: Enzyme-Linked-Immunosorbent-Assay
DNPH: Dinitrophenylhydrazine (DNPH)
FD: Fluorescein diacetate
PI: Propidium iodide
DMSO: Dimethyl sulfoxide
