# Supplementary material for: Non-invasive photobiomodulation treatment in an Alzheimer Disease-like transgenic rat model
Source: Theranostics. 2022 Feb 14;12(5):2205–31. doi: 10.7150/thno.70756 (PMC8899582; doi:10.7150/thno.70756)
Supplement: Supplementary file 1 — Supplementary figures. [file thnov12p2205s1.pdf]

# Supplementary Information

## Supplementary Figures

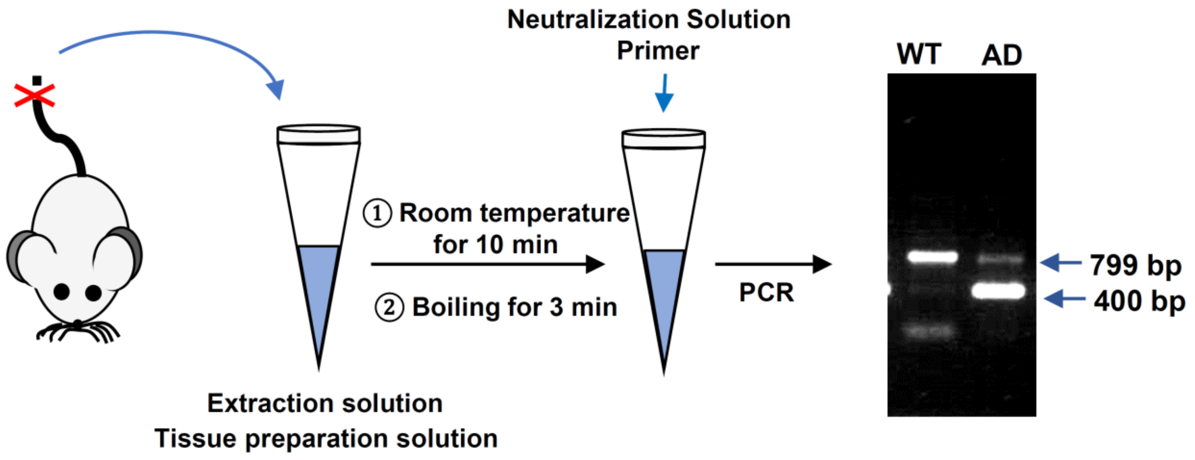


**Figure S1. Schematic diagram of genotyping.** A 400bp APP Tg product was displayed in samples from AD animals.

**A**

**Diagram of PBM treatment**

**Rats were restrained in a plastic cone**

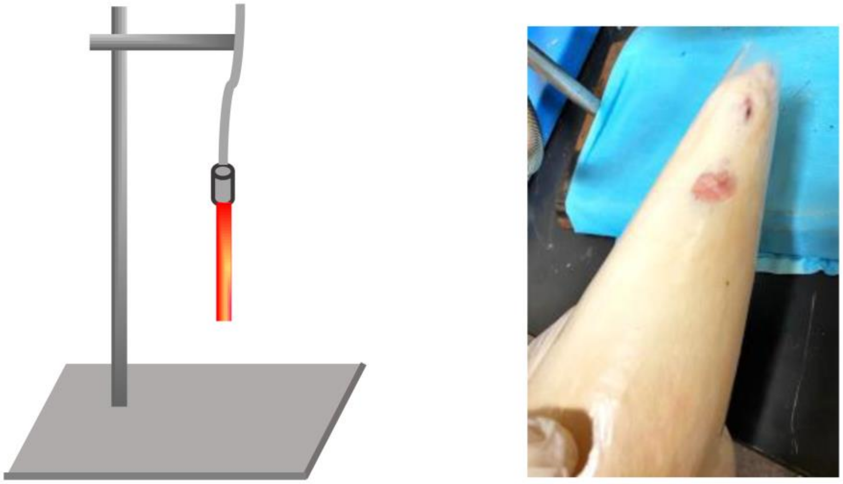

**B**

**Device Information**

| | |
|---|---|
| Model Identifier | MDL-III-808-500 mW |
| Emitter Type | Infrared Diode Laser |
| Wavelength (nm) | 808 $\pm$ 3.0 nm |
| Number of Emitters | 1 |
| Operating mode | Continuous Wave |
| Transverse mode | Round |
| Beam Delivery System | Fiberoptic |
| Beam Area | 1.5 $cm^2$ |
| Irradiance | 350 $mW/cm^2$, Scalp<br>25 $mW/cm^2$, Cortex |
| Number of Treatments | Multiple |

**Figure S2. PBM treatment and device information. (A)** The distance between the laser tip and the animal's scalp was adjusted to 35 cm to generate a 1.5 $cm^2$ laser spot on the animal's scalp. Rats were restrained in a plastic cone when receiving PBM treatment. **(B)** Device information.

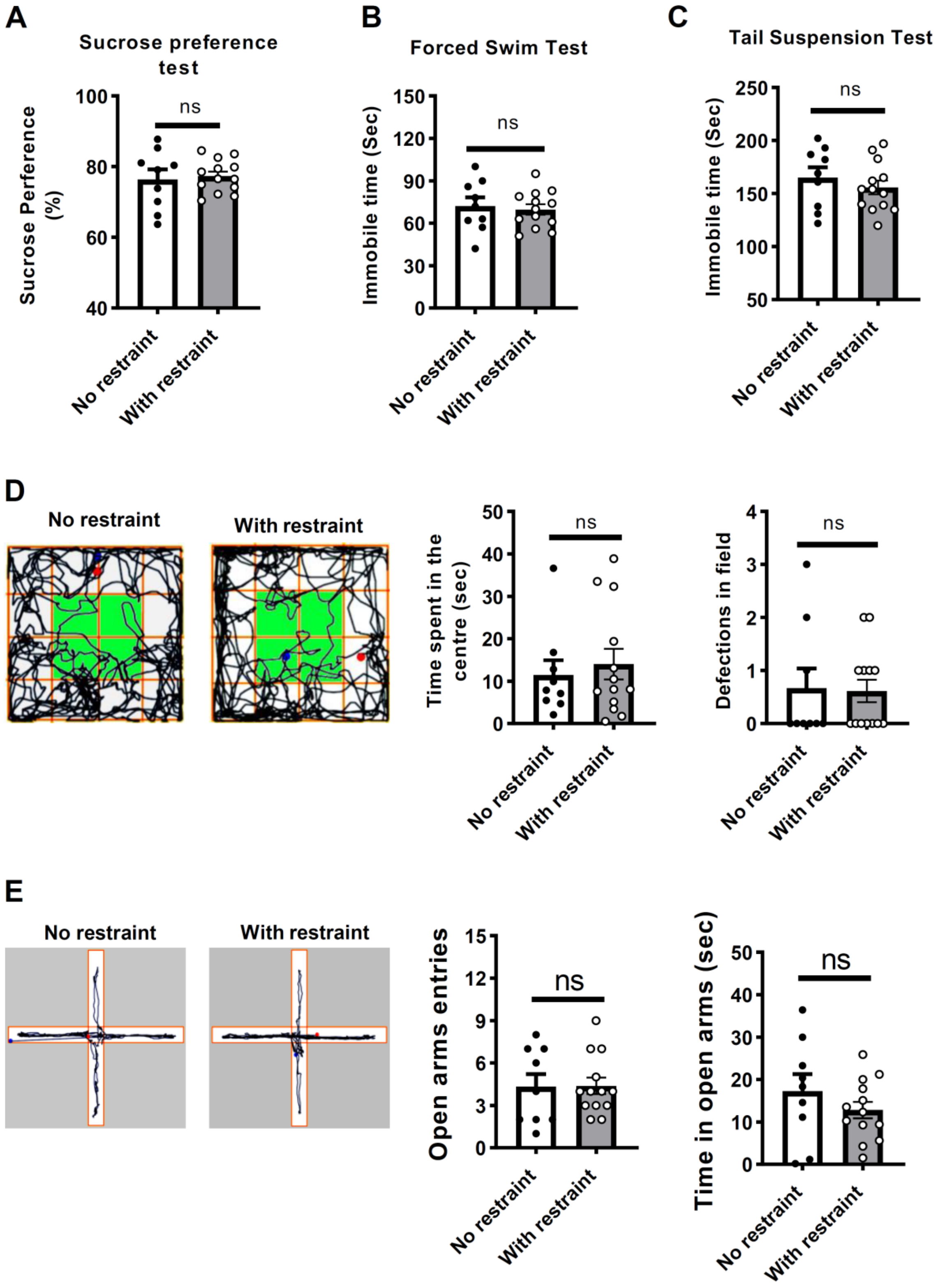
A
Sucrose preference test
ns
Sucrose Perference (%)
100
80
60
40
No restraint
With restraint
B
Forced Swim Test
ns
Immobile time (Sec)
150
120
90
60
30
0
No restraint
With restraint
C
Tail Suspension Test
ns
Immobile time (Sec)
250
200
150
100
50
No restraint
With restraint
D
No restraint
With restraint
ns
Time spent in the centre (sec)
50
40
30
20
10
0
No restraint
With restraint
ns
Defections in field
4
3
2
1
0
No restraint
With restraint
E
No restraint
With restraint
ns
Open arms entries
15
12
9
6
3
0
No restraint
With restraint
ns
Time in open arms (sec)
50
40
30
20
10
0
No restraint
With restraint

**Figure S3. Restraint in PBM treatment did not induce anxious-depressive-like behavior.** The sucrose preference test (A), forced swim test (B), tail suspension test (C), open field test (D), and elevated plus maze were performed to measure anxious-depressive-like behavior. Data are presented as mean ± SEM (n=9-13). ns indicates no significant difference (*P* > 0.05).

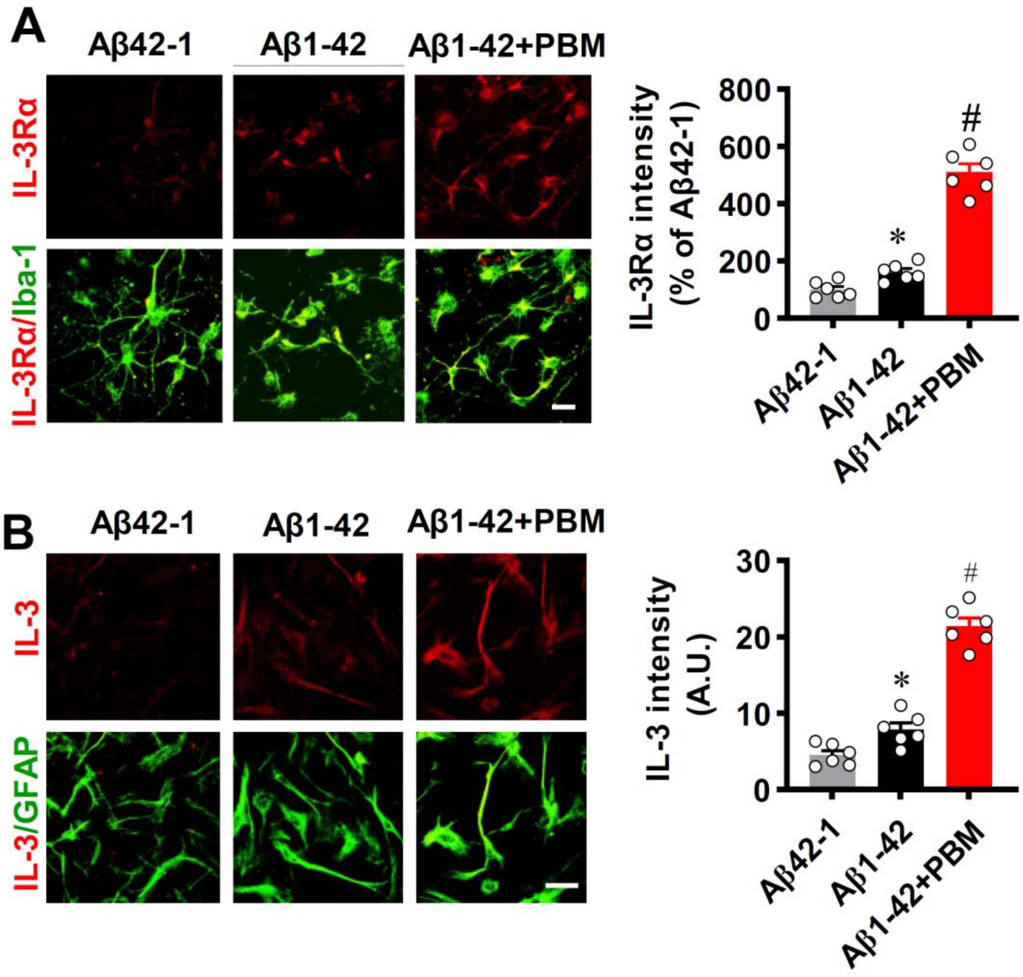


**Figure S4. PBM treatment regulates the expression of microglial IL-3Rα and astrocytic IL-3 in vitro**

**(A)** Representative confocal microscopy images of IL-3Rα (red) and Iba-1(green). The relative fluorescent intensity was analyzed and presented as a percentage change versus sham. **(B)** Representative confocal microscopy images of IL-3 (red) and GFAP (green). The relative fluorescent intensity was analyzed and presented as a percentage change versus sham. Data are

presented as mean ± SEM (n = 6). Scale bar = 20 μm. Data are presented as mean ± SEM (n = 6). *$P$ < 0.05 versus WT group, $^{\#}P$ < 0.05 versus AD group.

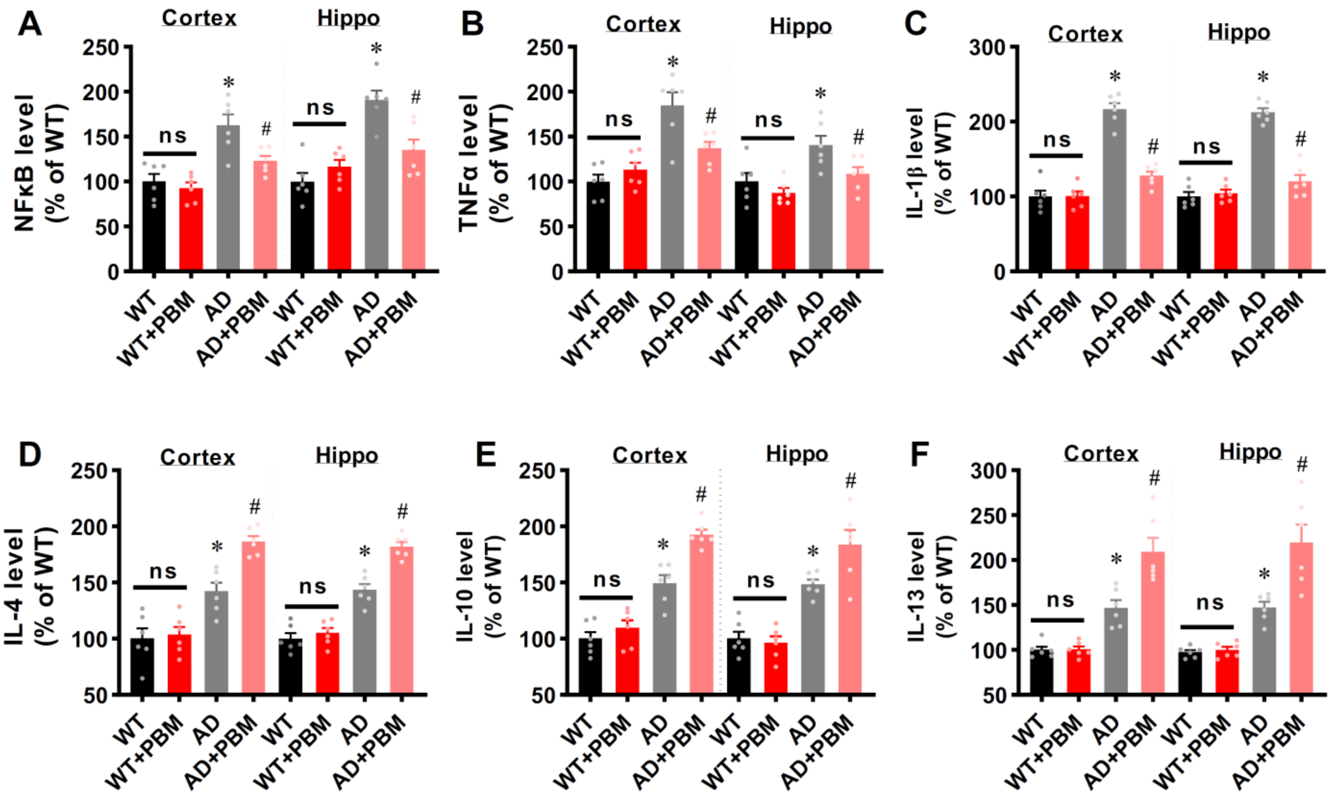


**Figure S5. PBM treatment suppresses the release of pro-inflammatory factors and promotes the release of anti-inflammatory factors**

Inflammatory ELISA assay kits were applied to detect the level of pro-inflammatory factors (i.e., NFκB) **(A)**, TNF-α **(B)**, and IL-1β **(C))** and anti-inflammatory factors (i.e., IL-4 **(D)**, IL-10 **(E)**, and IL-13 **(F)).** Data are presented as mean ± SEM (n = 6). *$P$ < 0.05 versus WT group, $^{\#}P$ < 0.05 versus AD group. ns indicates no significant difference ($P$ > 0.05).

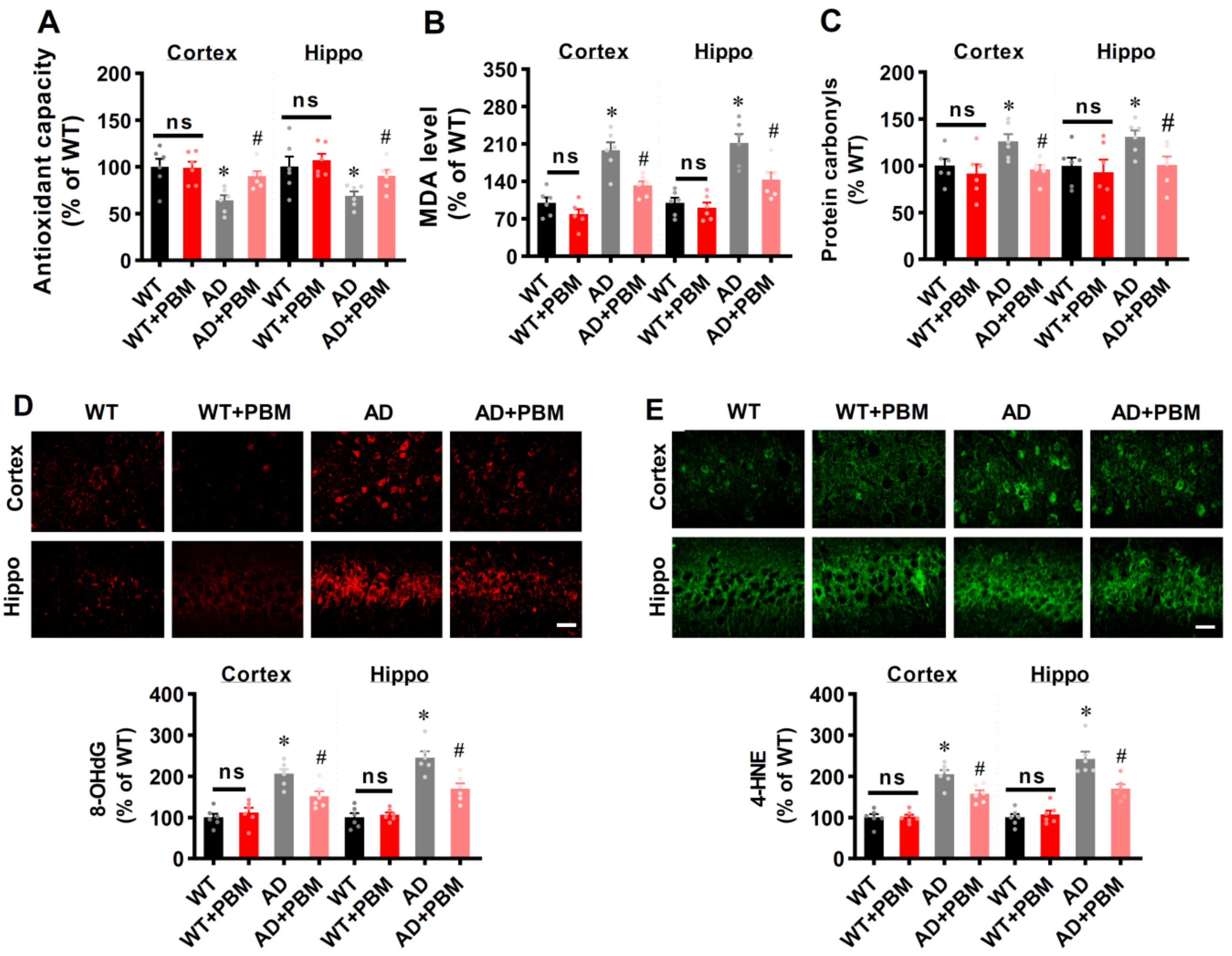


**Figure S6. PBM treatment alleviates oxidative damage in the cortex and hippocampus**

**(A)** The total antioxidant capacity, **(B)** MDA levels, and **(C)** protein carbonyls were measured using protein from both the cortex and hippocampus using corresponding assay kits. Data were quantified as percentage changes versus the WT group and expressed as presented as mean ± SEM (n = 6). **(D)** Representative confocal microscopy images of 8-OHdG (a biomarker of oxidative DNA damage) and 4HNE (a lipid peroxidation marker). The immunotolerance intensities of 8-OHdG and 4HNE were quantified as percentage changes versus the WT group. Scale bar = 20 μm. All data are presented as mean ± SEM (n = 6). **P* < 0.05 versus WT group, # *P* < 0.05 versus AD group. ns indicates no significant difference (*P* > 0.05).

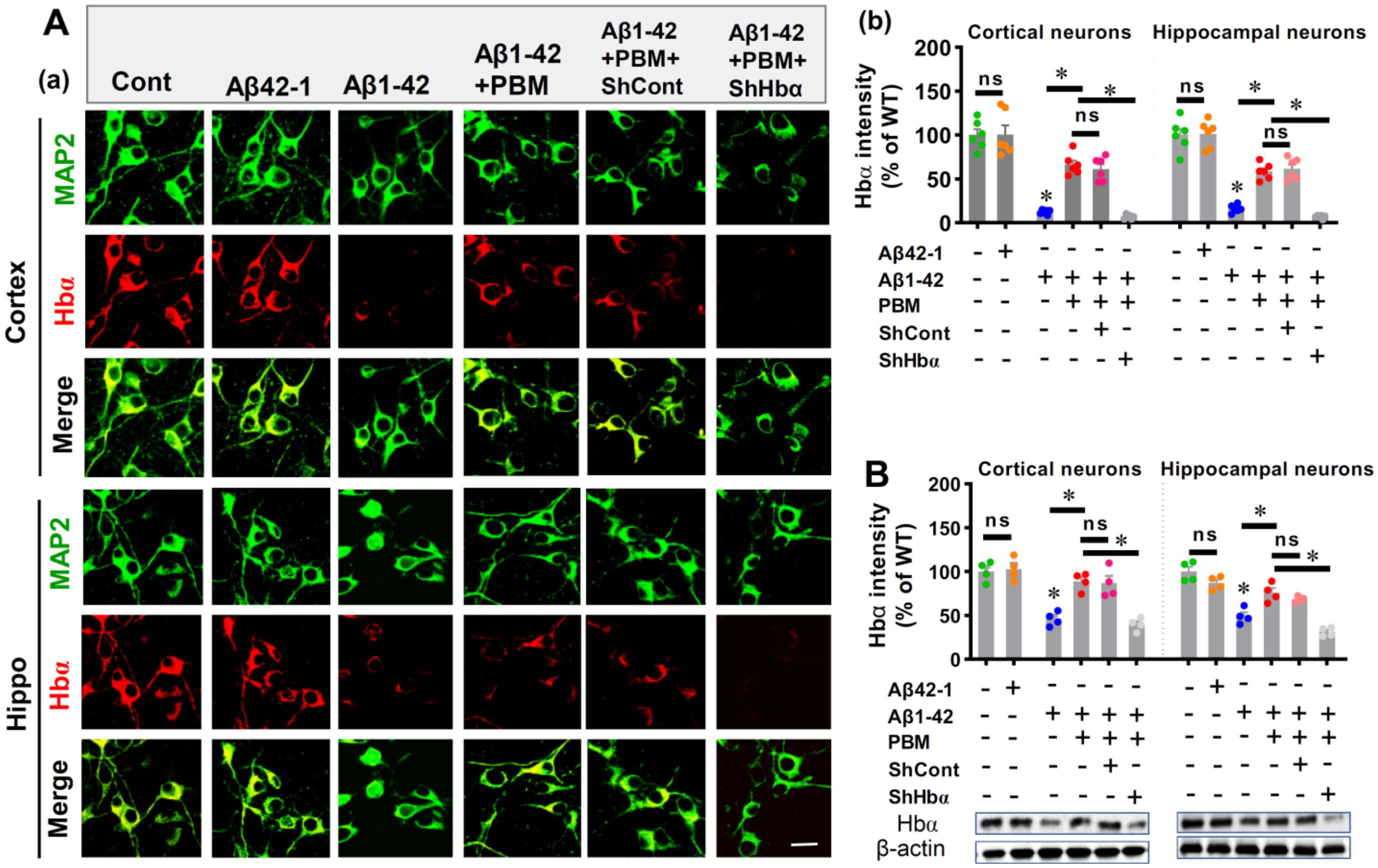


**Figure S7. PBM treatment preserves neuronal hemoglobin in vitro**

**(A)** The primary culturing neurons from both the cortex and hippocampus were stained with MAP2 (green) and hemoglobin α (red, HBα) (a). The immunofluorescence intensity of HBα was quantified as percentage changes versus the cont. group and expressed as presented as mean ± SEM (n = 6). Scale bar = 10 µm. **(B)** Western blotting and quantitative analyses of HBα in the neurons. The intensity of HBα was measured using Image J software and expressed as quantified as percentage changes versus the cont. group. Data are expressed as mean ± SEM (n = 4). *$P < 0.05$. ns indicates no significant difference ($P > 0.05$).

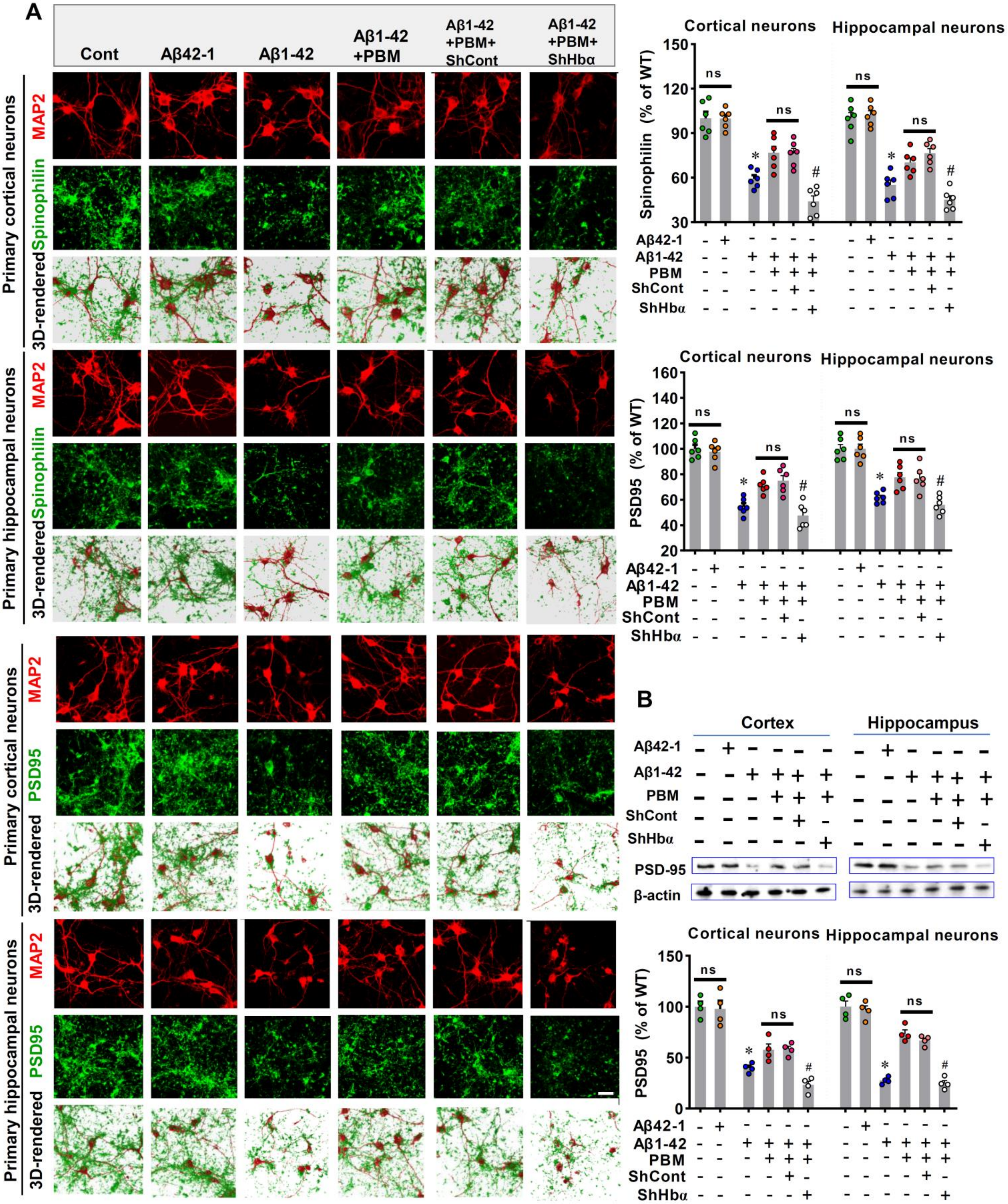
A
Cont
Aβ42-1
Aβ1-42
Aβ1-42 +PBM
Aβ1-42 +PBM+ ShCont
Aβ1-42 +PBM+ ShHbα
Primary cortical neurons
Primary hippocampal neurons
MAP2
Spinophilin
PSD95
3D-rendered
Cortical neurons
Hippocampal neurons
Spinophilin (% of WT)
PSD95 (% of WT)
ns
Aβ42-1
Aβ1-42
PBM
ShCont
ShHbα
B
Cortex
Hippocampus
PSD-95
β-actin

**Figure S8. Hbα knockdown abolishes PBM treatment's effects on pre/post-synaptic proteins.** **(A)** Representative confocal microscopy images of MAP2 (red) with Spinophilin or PSD-95 (green). Scale bar = 20 µm. **(B)** Western blotting and quantitative analyses of PSD-95. Data were presented as a percentage change versus the control group (n=8). *$P < 0.05$ versus control group, # $P < 0.05$ versus PBM group. ns indicates no significant difference ($P > 0.05$).

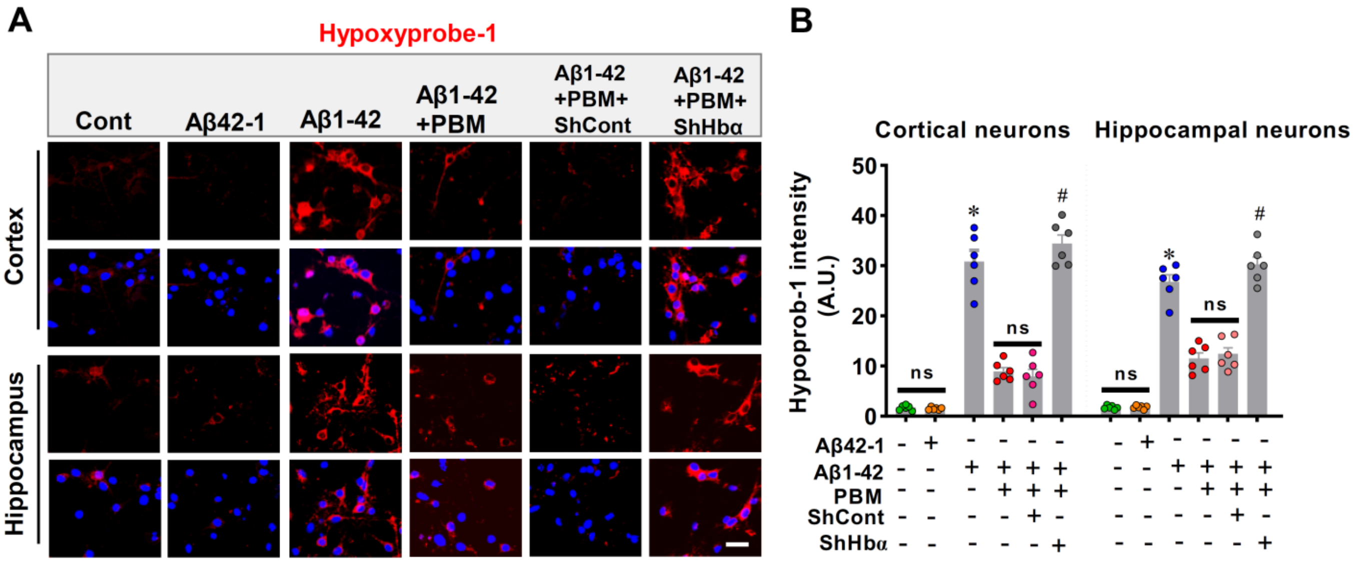


**Figure S9. PBM treatment alleviates Aβ1-42 induced neuronal hypoxia in vitro**

**(A)** Representative confocal microscopy images of neurons stained hypoxyprobe-1 kit. **(B)** The immunofluorescent intensities of hypoxyprobe-1 in the culturing neurons from both the cortex and hippocampus. Scale bar = 10 µm. Data are expressed as mean ± SEM (n = 6). *$P < 0.05$ versus cont. group, # $P < 0.05$ versus PBM group. ns indicates no significant difference ($P > 0.05$).
